# Improving reporting standards in quantitative educational intervention research: introducing the CLOSER and CIDER checklists

**DOI:** 10.1007/s44322-024-00022-9

**Published:** 2025-02-03

**Authors:** Rebecca Upsher, Eleanor Dommett, Sophie Carlisle, Sarah Conner, Geraldene Codina, Anna Nobili, Nicola C. Byrom

**Affiliations:** 1 Department of Psychology, Institute of Psychiatry, Psychology and Neurosciences, King’s College London, London, UK; 2 Health Service and Population Research, King’s College London, London, UK; 3 Transforming Assess and Student Outcomes in Higher Education, London, UK; 4 Institute of Education, University of Derby, Derby, UK; 5 Student Wellbeing Support, University of Derby, Derby, UK

**Keywords:** Educational interventions, Reporting standards, Research methodology, Quantitative research, Checklist development, Evidence-informed practice

## Abstract

In educational research, the complexity of interventions and the diversity of contexts challenge the adequacy of existing reporting standards, primarily tailored for health interventions and randomised controlled trials. Recognising the critical need for appropriate tools to support education research, we developed two checklists: the CheckList Of Standards of reporting in Education Research (CLOSER) and the Checklist for Intervention Description of Education Research (CIDER). CLOSER was designed to guide the comprehensive reporting of quantitative educational intervention research from the abstract to the discussion for multiple study designs. Concurrently, CIDER details the precise features of educational interventions. The final refinements of CLOSER and CIDER comprise of 34 and 17 items, respectively, developed through a five-stage process, including tool adaptation, expert feedback, a 2-round modified Delphi consensus survey, and final refinements. These checklists aim to enhance the clarity of educational intervention research findings, facilitating their replicability and subsequent meaningful translation into practice and policy. This addresses the gap in educational research reporting standards and sets the stage for a more robust, evidence-informed approach to educational intervention development and evaluation.

**Supplementary Information:**

The online version contains supplementary material available at 10.1007/s44322-024-00022-9.

## Introduction

Boykin (1972) argued for the need for research in education, emphasising its crucial role in the development of educational practices. High-quality research, uniquely positioned to provide valid and insightful accounts of educational realities, serves as a reliable basis for decision-making by policymakers (Boykin, 1972; Edovald & Nevill, 2021). As the demand for evidence-based policy intensifies, the necessity for robust evidence supporting education-based interventions becomes even more pronounced. This evidence, grounded in research quality assessed by criteria such as validity, reliability, and dependability (Hodkinson, 2004; Winch et al., 2015), guides principals, policy, and practice in the field of education.

Checklists to guide research and reporting practices enhance the reliability and replicability of research, with journals that endorse them demonstrating higher reporting quality than those that do not (Anderson et al., 2021). The most widely utilised checklists for research reporting are found in the field of health research, such as the Consolidated Standards of Reporting Trials (CONSORT) checklist and the Template for Intervention Description and Replication (TIDieR) checklist. The CONSORT checklist is designed to enhance the reporting of randomised controlled trials (RCTs), thereby standardising the process and ensuring comprehensive and transparent documentation (Moher et al., 2012). The TIDieR checklist aims to improve the reporting of health interventions, facilitating their replication by providing a template for researchers to detail the necessary components of an intervention (Hoffmann et al., 2014). However, neither the CONSORT nor the TIDieR checklists are appropriate for education intervention research. For example, the CONSORT checklist has a narrow focus on RCTs only and, therefore, is unsuitable for the range of quantitative study designs employed in educational research, including RCTs, quasi-experimental, pre-post, and cross-sectional studies. The TIDieR checklist is too generic in scope, creating challenges for reporting educational interventions (Dijkers, 2021).

Analogous tools in educational research include the Guideline for Reporting Evidence-Based Practice Educational Interventions and Teaching (GREET) checklist (Phillips et al., 2016), Standards for QUality Improvement Reporting Excellence in Education (SQUIRE-EDU) (Ogrinc et al., 2019), and Defined Criteria To Report Innovations in Education (DoCTRINE) (Blanco et al., 2022). However, these tools focus specifically on educational interventions in the fields of health, medical, and clinical education (Blanco et al., 2022; O’Brien et al., 2020), limiting their broader applicability in education research. Despite the availability of these tools, research on educational interventions often suffers from insufficient reporting of intervention characteristics, population information, crucial study details, and basic statistical and analytical data (Abulfaraj et al., 2024; Albarqouni et al., 2018; Meinema et al., 2019; Upsher et al., 2022). Poor reporting reduces the quality, replicability, and reliability of research, hampering progress in the field by limiting the ability to draw comparisons between studies or synthesise data (Altman, 2015; Chalmers & Glasziou, 2009; Upsher et al., 2022).

In educational intervention research, replicating study methods and outcomes is crucial, yet it must accommodate the diverse cultural contexts, pedagogies, and methodologies involved (Albarqouni et al., 2018). Effective replication requires thorough reporting to adapt interventions for different settings, balancing fidelity to core principles with the flexibility for context-specific adjustments. This not only facilitates educators in applying research to practice but also supports policy-making and curriculum development, aiming to improve a variety of educational outcomes. Therefore, there is an urgent need for new checklists that are widely applicable to a range of educational study designs and contexts.

For reporting checklists to be successful, they must be relevant to the field and acceptable to researchers (Hohmann et al., 2018). Thus, the objective of this research was to leverage expert consensus in developing innovative checklists designed to (a) support the reporting of educational intervention research across multiple quantitative study designs and (b) ensure replicable reporting of educational interventions in a range of educational settings, including schools, further education, higher education, and beyond health education alone.

## Methods

This research led to the formulation of two checklists, one for reporting the entire research process in education intervention research and the other for reporting the features of educational interventions:

- CheckList Of Standards of Reporting in Education Research (CLOSER): This checklist guides the reporting of quantitative education intervention research studies, spanning from the abstract to the discussion section of a journal manuscript. CLOSER is designed for use across various quantitative study designs that evaluate educational interventions, including RCTs, quasi-experimental designs, pre-post studies, and cross-sectional studies.
- Checklist for Intervention Description of Education Research (CIDER): This checklist was developed to help researchers detail the features of educational interventions. It can be applied within the methods section of both quantitative and qualitative manuscripts. When dealing with quantitative studies, it serves to complement the methods section of the CLOSER checklist.

These checklists were developed through a five-stage process outlined below.

- Stage 1: Tool adaptation

Two researchers (RU and AN) initially reviewed existing checklists for reporting interventions, predominately the CONSORT (Moher et al., 2012) and TIDieR (Hoffmann et al., 2014), and adapted them, as needed, to suit education intervention research and multiple quantitative study designs. This adaptation generated the first drafts of the CLOSER and CIDER checklists (refer to supplementary materials, S1).

- Stage 2: Expert team development

The checklists were enriched by the interdisciplinary expertise, spanning education, health, and psychology, of seven team members (RU, AN, ED, SC, SC, GC, NB), who have experience in evidence synthesis, as well as qualitative and quantitative methodologies. This facilitated the bridging of paradigms, making quantitative research more accessible to qualitative researchers, and breaking down silos across fields such as education and health.

The team provided individual written feedback on the checklist items from Stage 1. This feedback was collated and discussed in two online meetings with the team. Based on the written feedback and subsequent discussion, the checklists were further refined (see supplementary materials, S2).

- Stage 3: Delphi consensus survey round 1 method and results

An online modified Delphi consensus survey method (Murphy et al., 1998) was deployed using the surveying platform Qualtrics to solicit feedback from a broader group. Members of international editorial boards of education journals were invited to provide their expert input.

In Delphi Round 1, 28 participants engaged in this process. Their demographic information is reported in Table 1. These participants came from a variety of academic disciplines, roles, and countries, with a predominant representation from the 'Education and Teaching' academic field. Participants were keen to see these checklists published in a range of places, including journal articles endorsed by journals in author guidelines, university library resources, ethical guidelines, educational online platforms, and study preregistration websites. Most participants reported ‘often’ reading educational research studies (*n* = 22, 79%) and conducting education research (*n* = 15, 54%).

**Table 1:** Demographics of Delphi participants

Demographics	*N* (%)
**Gender **	
Female	14 (56.0)
Male	11 (44.0)
**Ethnicity**	
White	23 (92.0)
Indian	1 (4.0)
Prefer not to say	1 (4.0)
**Academic role **	
Professor	8 (33.3)
Managerial^a^	6 (25.0)
Postgraduate Research	4 (16.7)
Associate Professor	3 (12.5)
Teaching Fellow/ Lecturer/ Senior Lecturer	3 (12.5)
**Subject discipline**	
Education and Teaching	15 (60.0)
Psychiatry, Psychology & Neuroscience	8 (32.0)
Health and Social Care	1 (4.0)
Computer Science	1 (4.0)
**Country of work**	
United Kingdom	10 (43.5)
Australia	4 (17.4)
United States	3 (13.04)
Italy	1 (4.35)
New Zealand	1 (4.35)
Norway	1 (4.35)
South Africa	1 (4.35)
Multi	2 (8.70)

^a^including Academic Manager, director, deputy CEO, Head of Department

The CLOSER and CIDER checklists, with 38 and 17 items, respectively, at this stage, were examined. Participants were asked to rate each item as "omit", "desirable with edits", "desirable as is", "essential with edits", and “essential as is” and provide qualitative responses detailing suggestions for improvement. In this round, CLOSER checklist items were most frequently rated as “essential”, “essential with edits”, or “desirable”. For the CIDER checklist items, all were most frequently rated as “essential”. Therefore, no items were omitted based on quantitative survey responses. Qualitative feedback from participants and consultation with the expert team prompted adjustments, removal of items and new item additions for the second Delphi round (see supplementary materials, S3).

- Stage 4: Delphi consensus survey round 2 method and results

In Delphi Round 2, 19 participants (from Stage 3) reviewed the modified CLOSER and CIDER checklists, now with 38 and 18 items respectively. Participants either approved the items for inclusion in the final checklist or stated their objections, again providing qualitative general feedback as well. The majority approved the inclusion of all items in the final versions of both checklists (see supplementary material, S4).

- Stage 5: Final refinements of checklists

The feedback from Delphi Round 2, coupled with further consultation with the expert team, led to additional refinements and the finalisation of the CLOSER (Table 2) and CIDER (Table 3) checklists, which contain 34 and 17 items, respectively.

- The CLOSER checklist explanation and elaboration

**Table 2:** The CLOSER checklist

Item 1. Abstract: Structured summary of design, methods, results, and conclusions.
Item 2a. Introduction (Background and objectives): Background and explanation of rationale for study.
Item 2b. Introduction (Background and objectives): Succinctly state: (i) overall aim of the intervention, (ii) any specific objective(s), (iii) study hypotheses or research questions.
Item 3a. Methods (Study Design): Description of study design (such as randomised controlled trials, quasi-experimental, pre-post studies, or cross-sectional).
Item 3b. Methods (Study Design): State any changes to methods after the study started (such as eligibility criteria), with reasons.
Item 3c. Methods (Study Design): State whether ethical clearance has been obtained for this research.
Item 4a. Methods (Participants): State eligibility criteria for participants at (i) group (e.g., cohort type) level. (ii) individual level (e.g., demographic characteristics).
Item 4b. Methods (Participants): Report dates defining the periods of recruitment and follow-up.
Item 4c. Methods (Participants): (i) Report methods of recruitment. (ii) Provide details on whether participants who met the eligibility criteria were given equal opportunity to participate.
Item 5a. Methods (Interventions): Summarise the intervention with sufficient details to implement in other contexts. Please use the CIDER checklist here.
Item 5b. Methods (Interventions): Where applicable, provide sufficient details of control condition(s) to allow their implementation in other contexts.
Item 6a. Methods (Outcomes): Outline pre-specified primary and secondary outcome measures, including (i) how and when they were assessed. (ii) report whether they were validated and/or reasons for including unvalidated measures.
Item 6b. Methods (Outcomes): Report any changes to outcomes after the study commenced, with reasons.
Item 7a. Methods (Sample size): State how the sample size was determined.
Item 7b. Methods (Bias): Identify potential sources of bias. Indicate any efforts to address potential sources of bias.
Item 7c. Methods (Similarity of interventions): If relevant, provide a description of the similarity of interventions.
For researchers undertaking a randomised controlled trial, follow items 8 and 9. Otherwise please skip to item 10.
Item 8. Methods (Sequence generation): Report (i) the method used to generate the random allocation sequence; (ii) the type of randomisation (e.g., computer-generated, quasi-random, etc.); (iii) details of any restriction (such as blocking and block size).
Item 9. Methods (Allocation concealment and implementation): Report (i) who generated the random allocation sequence, (ii) who enrolled participants, and (iii) who assigned participants to interventions.
Item 10. Methods (Blinding): If done, report who was blinded after assignment to interventions (e.g., participants, providers, those assessing outcomes) and how.
Item 11. Methods (Statistical methods): Report (i) statistical methods used to compare groups for primary and secondary outcomes, (ii) methods for additional analyses, such as subgroup analyses and adjusted analyses. State reason if subgroup analyses are not possible or not appropriate.
Item 12. Results (Participant flow): For each group, report the numbers of participants (i) who were assigned to or participated in each group, (ii) received intended intervention, (iii) were analysed for the primary outcome, and (iv) (where relevant), report losses and exclusions (after randomisation or group allocation where relevant), together with reasons.
Item 13. Results (Baseline data): Provide a table showing baseline demographic characteristics for each group.
Item 14. Results (Numbers analysed): Report the numbers analysed for each group (where applicable) and overall cohort.
Item 15a. Result (Outcomes and estimation): For each primary and secondary outcome, report (i) the results for each group, at each timepoint. (ii) the estimated effect size and its precision (such as 95% confidence interval).
Item 15b. Results (Outcomes and estimation): For binary outcomes, present both absolute and relative effect sizes.
Item 16. Results (Ancillary analyses): Report the results of any other analyses performed, including subgroup analyses and adjusted analyses.
Item 17. Results (Harms): Report any unintended harms or effects in each group. Indicate how this was handled.
Item 18. Discussion (Generalisability): Comment on the ability of study findings to be implemented in other contexts.
Item 19. Discussion (Generalisability): Comment on the ability of study findings to be implemented in other contexts.
Item 20. Discussion (Limitations): State study limitations.
Item 21. Other information (Registration): State registration number and name of study registry.
Item 22. Other information (Protocol): Report where the full study protocol can be accessed, if available.
Item 23. Other information (Funding): State the sources of funding and other support, the role of funders.
Item 24. Other information (Data repository): Report the use of any data repository.

**Table 3:** The CIDER checklist

1 Brief title of intervention: Provide the name or a phrase that captures key 'essence' or intention of the intervention.
2 Aim and objectives of intervention: Succinctly state: (i) overall aim of the intervention. (ii) objective(s) and/or intended learning outcome(s) of the intervention.
3 Rationale for intervention: Report the purpose of intervention development with reference to relevant theory or observations.
4 Participants: (i) Report who the intervention was developed for. (ii) Report if the intervention was open to all participants across the institution or for a specific cohort. (iii) Report any collected participant demographic data such as age, gender, ethnicity, socioeconomic status.
5a Intervention provider(s): (i) Indicate who provided the intervention, e.g., state the job title/role of instructor(s) and the education organisation providing the intervention. (ii) report the relationship between intervention provider and developer, (iii) Comment on any prior relationship between participants and instructors. (iv) Report the instructors’ expertise, professional discipline, background and any relevant training they have undertaken.
5b Intervention developer(s): (i) Report who designed the intervention (developer[s]) and their professional background(s) and expertise. (ii) Report whether developer(s) were also involved in delivering the intervention (instructor[s]) and/or trained the instructors. (iii) Report whether participants (particularly those with lived experience where relevant) were involved in co-creation of the intervention. If so, explain their involvement, if not, provide reasoning.
6a Institutional setting: Indicate whether the intervention was conducted across one or multiple institutions. For each institution, report their nature and location, e.g., the nature of the institution (i) Urban or rural; (ii) School, further education, higher education; (iii) Teaching on campus, online or both. Report (iv) the size of the institution, i.e. total student population.
6b Intervention location: (i) Report the location(s) where the intervention occurred, e.g., classroom, lecture theatre, online, workplace placement, off-campus visits, green space. (ii) Include relevant infrastructure details, e.g., size of space, formal or informal layout.
7 Intervention timing and duration: Indicate (i) The number of times the intervention was delivered, and over what period of time. (ii) The number of sessions (and any home practice where relevant). (iii) If the content was self-paced, how long the content was designed to be completed in (e.g., 30 hours across two semesters), and what guidance was given to the participants. (v) The time of year/semester the intervention was delivered, including any other time-appropriate information, e.g., delivered close to the exam period..
8a Delivery – Modes and content: (i) Briefly outline main delivery method(s) for the intervention, e.g. face-to-face lectures, seminars, workshops; online videos, discussion. (ii) State whether activities were individual or group-based; instructor-led or self-paced by participants. (iii) Provide an outline of main content for each session or activity. (iv) Report the ratio of participants to instructors.
8b Delivery – Procedures: Indicate whether the intervention was (i) Embedded in the core curriculum, e.g., formally taught, included in assessment design, aligned with pedagogical approach; or (ii) Run outside of the core curriculum. If the intervention was curriculum embedded, indicate whether the intervention was credited, compulsory, and/or assessed (add details of assessment if relevant). Report the nature of any ‘home practice’ participants were given. (ii) Report any incentives offered to student participants.
9 Materials: Report any physical or informational materials used in the intervention, including those: (i) provided to participants; (ii) used in intervention delivery; (ii) used in training of intervention instructors. Indicate where the materials can be accessed.
10a Evaluation – Attendance: Indicate: (i) The number of participants who participated in the intervention. If the intervention included multiple sessions, indicate participant numbers for each one, and total of participants who completed the whole intervention, or dropped out mid-way. (ii) How attendance figures were recorded, and by whom. (iii) Any strategies that were used to facilitate attendance.
10b Evaluation – Delivery: (i) Report any form of observation or assessment of the intervention delivery, i.e., instructor observation, student satisfaction questionnaire.
10c Evaluation – Intervention design: Indicate the extent to which the intervention was delivered as planned with regard to: (i) Participants, e.g., whether they constituted a representative demographic sample of the institution’s student cohort. (ii) Intervention materials. (iii) Procedures. (iv) Instructors. (v) Mode of delivery. (vi) Location and setting. (vii) Number of sessions and their frequency. (viii) Timing and duration of the intervention.
10d Evaluation – Outcome measurements: Indicate whether the outcome of the intervention was directly measured, e.g., in a stress management intervention, was stress measured pre and post-intervention.
11 Modifications to intervention: If the intervention design was modified during the study, indicate the changes (what, why, when, and how) and the reasons for the changes.

Below, we enumerate each item in the CLOSER checklist, providing a comprehensive explanation and some examples. Please note that the examples are for illustration purposes only and should not be replicated exactly. Each study write-up will naturally vary depending on the context and design of the study.

- Item 1. Abstract: Structured summary of design, methods, results, and conclusions.

An abstract provides a concise and clear summary of the research paper, making it easier for readers to quickly understand the study's key elements and relevance. In a structured abstract, distinct sections are dedicated to the study's background, objectives, study design, methods, results, and conclusions.

- Item 2a. Introduction (background and objectives): Background and explanation of rationale for study.

The background and rationale sections of a research manuscript contextualise and justify the research, providing a clear understanding of the study's origins, its placement within the broader academic conversation, and its contribution to the field. The background offers a concise overview of what is already known in the area of study, highlighting existing research, key findings, and any evidence gaps or inconsistencies. Drawing from this background, the rationale then outlines why the study is both timely and essential, and explains why addressing a particular research question or gap is important.

- Item 2b. Introduction (background and objectives): Succinctly state: (i) overall aim of the intervention, (ii) any specific objective(s), (iii) study hypotheses or research questions.

The introduction also assists readers in understanding the researchers’ aim(s). The overall aim of the intervention summarises the broad goal of the study, i.e., what the intervention seeks to change or improve. Specific objectives refer to measurable and time-bound targets that contribute towards achieving the overall aim. The study hypotheses or research questions provide a clear direction for the study, indicating what the researchers plan to investigate or predict.

Example

This research introduces a literacy programme, utilising tablet technology, designed with the aim of improving reading comprehension in Year 5 students (aged 9–10). The research question is: 'Does a tablet-based literacy programme significantly improve reading comprehension and reading frequency compared to traditional methods?' The research objectives are to measure the difference in reading test comprehension scores and students' reading frequency from the first to the last week of the academic year. The hypothesis is that students utilising the tablet-based programme will exhibit superior a) reading comprehension and b) reading frequency than those who do not.

- Item 3a. Methods (study design): Description of study design (such as RCTs, quasi-experimental, pre-post studies, or cross-sectional).

The study design outlines the structure of the research and how it has been conducted, to enable readers to understand the validity of the research findings. Study design selection in education intervention research is driven by considerations such as time and budget constraints, ethical issues, and implementation feasibility. Design decisions must balance the use of rigorous methods with adaptability to real-world conditions, ensuring findings are robust and relevant. Below are definitions of the main quantitative study designs education intervention research may employ (others exist within these broader categories).

- RCTs are experimental designs where individuals are randomly allocated to an intervention or control group (Styles & Torgerson, 2018). The process of randomisation assists in reducing selection bias, with the objective of making the groups comparable.
- Quasi-experimental designs (or non-randomised controlled trials) involve an intervention and control group but do not have a random allocation of individuals to these groups (Gopalan et al., 2020).
- In pre-post studies, data are collected from the same group of individuals before and after an intervention, and there is no control condition (Marsden & Torgerson, 2012). The aim is to measure the effect of the intervention over time within the same group.
- In cross-sectional studies, data is collected from a defined population at a specific point in time, e.g., after an intervention (Spector, 2019). This design might compare different conditions e.g., intervention versus control.

Example

A pre-post study design to assess the impact of a new gamified learning application on the English vocabulary acquisition of Year 7 (aged 11–12) students was implemented. Students' vocabulary was measured before and after a term (10 weeks) of using the application during English lessons.

- Item 3b. Methods (study design): State any changes to methods after the study started (such as eligibility criteria), with reasons.

Including information regarding any changes to the methods after study commencement promotes transparency and integrity in research reporting, allowing for assessment of potential impact on the study’s findings. Changes might include adjustments to the eligibility criteria, data collection methods, or analysis techniques.

Example

After the study was initiated, the eligibility criteria were broadened to include students who scored below average on the national English test rather than only those in the lowest decile. This change was made to increase the sample size and improve the generalisability of the findings.

- Item 3c. Methods (study Design): State whether ethical clearance has been obtained for this research.

This item calls for the declaration of ethical clearance, by whom and with a reference number. Ethical clearance implies the study has been reviewed and approved by an ethics committee, and that it meets the ethical standards of scientific research. Researchers might also report adherence to ethical guidelines for educational research, if appropriate (BERA, 2018).

Example

Ethical clearance for this study was granted by the Institutional Review Board of [NAME] University (Reference Number: [NAME]/123/2023).

- Item 4a. Methods (participants): State eligibility criteria for participants at (i) group (e.g., cohort type) level. (ii) individual level (e.g., demographic characteristics).

Eligibility criteria describes the inclusion and exclusion criteria, which are key determinants of who can participate in a study, ensuring the scope and limitations of the study are understood. Inclusion criteria specify the characteristics that potential participants must possess to be eligible for the study, while exclusion criteria outline characteristics that disqualify potential participants from inclusion. These criteria may encompass both individual-level and group-level factors. Individual-level criteria may include, but are not limited to, demographic variables (age, gender, ethnicity), health status, neurodiversity status, behaviours, class attendance, educational level, or other factors related to educational outcomes. Group-level criteria apply to larger units, such as schools or classes, for example, a school's funding status or a class's year level.

Example

To be included in this study, pupils had to be studying at a state school in the north of England, with at least 80% attendance for the prior term, and receiving free school meals. Individuals were excluded from taking part if they were receiving additional maths tuition outside of school.

- Item 4b. Methods (participants): report dates defining the periods of recruitment and follow-up.

This item involves stating the periods for participant recruitment and follow-up, giving context to the study's logistics and potential seasonal influences, and allowing for comparison with other studies. Though exact dates are not necessary, months or academic terms add context. Details of how these dates align with the academic calendar is also contextually useful, e.g., the academic year in the UK often starts in September, while the academic year in Australia starts in January. The recruitment period relates to when participants were enrolled in the study, and the follow-up period indicates how long participants were monitored after the intervention.

Example

Recruitment for this study commenced at the start of the academic year (September 2023) and concluded in October 2023. The follow-up period continued until the end of the academic year (July 2024).

- Item 4c. Methods (participants): (i) Report methods of recruitment. (ii) Provide details on whether participants who met the eligibility criteria were given equal opportunity to participate.

This item recommends explicit details on recruitment and sampling methods. Here are some definitions of different types of recruitment methods:

- Gatekeepers: Utilises individuals or organisations outside of the research team to access specific participants, such that the gatekeepers have authority over who is recruited to the study.
- Snowballing and social media: Leverages personal networks and digital platforms to reach wider and more diverse audiences, including hard-to-reach populations.
- Email lists and online bulletins: Employs direct and broad-based digital communications to engage potential participants through targeted messages.
- Virtual learning environments and educational events: Recruiting within digital and physical educational settings, capitalises on engaged audiences.
- Posters and public advertisements: Utilises visual materials in strategic locations to attract local participants.
- Conferences and professional gatherings: Engages with professionals and academics in relevant fields during events to recruit interested individuals directly.
- Research Participant Databases: Accessing pre-registered individuals interested in participating in research, facilitating quick and targeted recruitment.

Here are some definitions of different types of sampling (Berndt, 2020):

- Random sampling: every member of a population has an equal chance of being selected. It is often used when there is a large population and a need for a representative sample.
- Stratified sampling: the population is divided into subgroups, or 'strata', usually based on a specific characteristic (such as grade level or socioeconomic status). Then, random sampling is used within each subgroup.
- Cluster sampling: similar to stratified sampling, but in this case, entire groups (or 'clusters') are randomly selected. This is often used when it is difficult or costly to sample from an entire population, such as selecting entire schools or classrooms within a district.
- Quota sampling: a non-random sampling method where researchers choose participants based on specific characteristics to ensure the sample represents those characteristics in the population.
- Purposive sampling: another non-random method involving selecting participants based on specific characteristics or qualities. It is often used when needing to reach a targeted sample quickly and where sampling for proportionality is not the main concern.

This item also details the provision of equal opportunities to all potential participants. Equal opportunity implies that all individuals meeting the eligibility criteria were given sufficient time to consider their participation, and had access to the same information about the study. This transparency ensures fairness in the recruitment process, reduces selection bias, and strengthens the credibility of the study's findings. Where equal opportunity is not possible a reason should be provided for this.

Example

Emails were sent to all students enrolled in undergraduate Business courses (purposive sampling). The emails outlined the study's purpose, what participation involved, and the voluntary nature of the study. Two weeks were allowed for interested students to respond, ensuring sufficient time for consideration. Equal opportunity was ensured as all students meeting the eligibility criteria (enrolled in a Business course, undergraduate level) received the same information and had the same opportunity to participate.

- Item 5a. Methods (Interventions): Summarise the intervention with sufficient details to implement in other contexts. Please use the CIDER checklist here.

For this item, please refer to the CIDER checklist, which is designed to guide researchers in detailing the features of educational interventions. If the journal's word count limit doesn't allow for the inclusion of all intervention features, researchers should utilise appendices or supplementary data or refer to other publications to provide additional context. This is important because comprehensive reporting of the intervention supports the replication and adoption of successful strategies in other educational settings. It also aids in the analysis of the intervention's effectiveness and helps in identifying potential areas for improvement.

- Item 5b. Methods (Interventions): Where applicable, provide sufficient details of control condition(s) to allow their implementation in other contexts.

This item encourages a detailed explanation of the control conditions used in the study, if applicable. The control condition provides a baseline against which the intervention's effectiveness can be compared, potential confounding factors can be understood, and the intervention's outcomes can be interpreted. Again, researchers could utilise appendices or supplementary data or refer to other publications to provide additional context. The CIDER checklist can also be used here to describe control condition features.

- Item 6a. Methods (Outcomes): Outline pre-specified primary and secondary outcome measures, including (i) how and when they were assessed. (ii) report whether they were validated and/or reasons for including unvalidated measures.

The primary outcome is the main outcome that a research study is designed to assess. The secondary outcome(s) are additional outcomes of interest that are not as crucial to the fundamental research question but can provide further insights or examine the mechanisms underlying the primary outcome.

When it comes to the validity of measures, researchers need to ensure their chosen outcome measures accurately reflect what they intend to measure. The validity of a measure is often established through previous research, and information about a measure’s validity can be found in the original papers where the measure was introduced, in validation manuscripts or in subsequent research using the measure (Sullivan, 2011). Note that the validity of a measure is always context and sample-specific.

While using validated measures is ideal (Heron et al., 2023), it is also recognised that in some cases, particularly with novel interventions or unique populations, it might be necessary to adapt existing measures or develop new ones. If unvalidated measures are used, reasons should be given. These details are necessary for readers to fully comprehend and interpret the findings and for other researchers to replicate or build upon the study.

Example

The primary outcome was improvement in wellbeing, assessed at the end of the semester using the Warwick-Edinburgh mental well-being scale (WEMWBS; CITE), a survey validated in undergraduate students (CITE). The secondary outcome was self-efficacy in technical writing, measured using an adapted version of the General Self-Efficacy Scale (CITE). While not validated for this specific context, it was chosen due to its robustness in various settings and relevance to the educational goals of the course.

- Item 6b. Methods (Outcomes): Report any changes to outcomes after the study commenced, with reasons.

In some cases, adjustments might be necessary due to unforeseen issues or new insights. This item requests transparency regarding any alterations to the outcome measures after the study has started, as unreported changes can lead to biases in the results.

Example

The study originally aimed to measure the impact of a curriculum content change on undergraduate psychology students' class attendance. However, due to the unexpected shift to remote learning caused by COVID-19, the class attendance measure was replaced with a measure of engagement with online learning materials.

- Item 7a. Methods (Sample size): State how the sample size was determined.

In quantitative research, the required sample size is usually determined through a power calculation based on anticipated effect sizes, desired power, and alpha level (Calculator.net, 2023; Kadam & Bhalerao, 2010). However, it is also important to note that while a power calculation can provide an estimate for the required sample size, practical factors such as response rate based on past studies or availability of the target population may also influence the final sample size. In some situations, the sample size may be determined by the minimal sample available in the given context, i.e., the size of a department or year group. In these cases, power calculations can be useful for determining the effect size that can be detected given a set sample size. Furthermore, where studies are advertised online or on notice boards, it may not be possible to determine exactly how many people were invited to participate.

Example

Example 1: A power calculation was conducted. In order to have adequate power to detect a small to medium effect size (Cohen's d = 0.3), with an alpha level of 0.05 and power at 0.80, the analysis indicated that a total sample size of 176 was required. Consequently, recruitment was aimed at approximately 90 students per group (intervention and control) to allow for potential dropouts.

Example 2: This study utilised a quasi-experimental design, with the sample size being determined by the number of students enrolled in third-year sociology classes during the 2023 Autumn semester at a large urban university in France. The intervention was offered to the three sociology classes, each having a consistent enrolment of approximately thirty students, giving a total potential sample size of 90 participants. Due to the contextual limitations, the sample size was constrained by the size of the sociology department and the availability of the target population rather than a formal power calculation. Based on this sample size, a power calculation determined that a medium effect size of 0.4 could be detected.

- Item 7b. Methods (Bias): Identify potential sources of bias. Indicate any efforts to address potential sources of bias.

It is important to identify any potential sources of bias and find ways to mitigate them, to ensure that study results are as reliable and valid as possible. This might involve using statistical methods to control for potential confounding factors or designing the study in a way that minimises the chances of these biases occurring. This consideration becomes especially relevant when evaluating complex interventions via quantitative methods (Anderson & Shattuck, 2012; Brown, 1992; Craig et al., 2008). Common sources of bias in quantitative research may include (not an exhaustive list) (Meuli & Dick, 2018):

- Self-selection bias: when participants choose to take part in a study, which may result in a group of participants who are different in some way from those who chose not to participate. For example, they might be more motivated or have more free time.
- Measurement bias: when there is a systematic error in the way data are collected or measured, which can skew the results.
- Confounding variables: when another factor, apart from the intended focus, influences the results. For example, in a study looking at the impact of a new teaching method on students' grades, the students' prior academic ability could also affect their grades, and thus it would be hard to disaggregate prior academic ability from the impact of the teaching method.

Students with higher prior academic achievement might have been more likely to use the online tool, potentially confounding the results. Therefore, previous academic achievement was controlled for in the analysis.

Example

Students with higher prior academic achievement might have been more likely to use the online tool, potentially confounding the results. Therefore, previous academic achievement was controlled for in the analysis.

- Item 7c. Methods (Similarity of interventions): If relevant, provide a description of the similarity of interventions.

This item asks for a comparison of the interventions (including control groups) used in the study, specifically looking for their similarities. In a study with a control group(s), it is essential that the intervention and control conditions are distinct and non-overlapping, to ensure the validity of the study's findings (Creswell & Creswell, 2017). For instance, if students can attend both intervention and control conditions, it would be difficult to attribute observed differences to the intervention. This is why researchers should report their efforts to maintain clear boundaries between the intervention and control groups and describe how similar or dissimilar these interventions were.

Example

The intervention and control groups were both delivered by the same teacher, in the same setting and ran for the same duration. All students were blind to their condition. The control condition was delivered in the morning, the intervention was delivered in the afternoon on the same day. The content was similar for both groups, but the teaching method differed. The control group attended standard literature-searching workshops with no mention of generative artificial intelligence (GenAI), whereas the intervention group received GenAI training to support literature searching. Students could not attend both workshops. After post-intervention survey measures were taken, both groups received resources from the other workshops and information on the differences between the conditions.

At this point in the checklist, items 8 and 9 are followed only if the study design is an RCT. Otherwise, researchers can skip to item 10.

- Item 8 Methods (Sequence generation): Report (i) the method used to generate the random allocation sequence; (ii) the type of randomisation (e.g., computer-generated, quasi-random, etc.); (iii) details of any restriction (such as blocking and block size).

For RCTs, this item recommends explaining how the sequence for random allocation (sequence generation) was produced. There are various appropriate methods, for example, a random number table or computerised random number generator (Torgerson et al., 2013). The level of randomisation, whether at the individual level (each participant was randomly allocated) or at the group level (e.g., entire classes or schools were allocated randomly), is important as it affects the overall study design and conclusions that can be drawn. This information is vital for understanding the robustness of the study and its susceptibility to bias, and it also helps other researchers who might want to replicate the study.

The type of randomisation refers to the method used to create this sequence e.g., simple randomisation or stratified randomisation. The details of any restriction, such as blocking and block size, are important to report. Blocking is a technique used to ensure that the groups are of equal size and the block size refers to the number of assignments in each block. For instance, a block size of four in a study with two groups (intervention and control) means that for every four participants, two will be assigned to the intervention and two to the control. This ensures a balance in the number of participants in each group across the trial.

Example

Simple randomisation was conducted at the individual level within the cohort of university students participating in the study. Participant allocation was achieved through a computerised random number generator, ensuring a 1:1 allocation ratio for intervention and control groups, without stratification; this means for every participant allocated to the intervention group, one participant is allocated to the control group. To address potential imbalances in group sizes given the anticipated sample size and enhance allocation concealment, blocked randomisation with mixed block sizes of four and six was employed. For example, in a block of four, two participants were allocated to each group, and in a block of six, three participants were allocated to each group. This approach, especially the use of mixed block sizes, maintains the overall 1:1 allocation ratio while adding unpredictability to the allocation sequence. Such unpredictability prevents selection bias and ensures the concealment of future allocations.

- Item 9. Methods (allocation concealment and implementation): Report (i) who generated the random allocation sequence, (ii) who enrolled participants, and (iii) who assigned participants to interventions.

In the context of an RCT, allocation concealment refers to the practice of hiding which group a participant will be assigned to until the moment of assignment. This prevents bias in the assignment of participants to intervention or control groups, as researchers cannot influence the allocation process. Common methods for achieving allocation concealment include the use of sealed envelopes or a centralised, computer-based system. Describing this mechanism demonstrates the robustness of the study's methodology and allows for the replication of the procedure in future studies.

Stating who generated the random allocation sequence, who enrolled participants, and who assigned participants to interventions can help to demonstrate that the study was designed to minimise potential sources of bias. It offers transparency and provides the reader with an understanding of how mitigated bias was achieved.

Example

A computer-based randomisation system allocated participants to either the intervention or control condition, assigning a unique code for each. Before baseline survey measures were obtained, only one team member (AB), who did not know the participants and was not involved in delivering the intervention or control conditions, could access the allocation to avoid any potential bias. Until this point, neither the researchers delivering the intervention or collecting data nor the participants knew to which group participants had been allocated. The enrolment of participants into the study was carried out by research staff who were unaware of the participant allocation groupings (CD). Once participants completed the baseline assessments, the designated team member (AB) then retrieved the participants’ group assignment from the system ensuring all aspects of participant assignment were conducted without bias, maintaining the concealment of allocation until the appropriate stage.

- Item 10. Methods (Blinding): If done, report who was blinded after assignment to interventions (e.g., participants, providers, those assessing outcomes) and how.

Blinding, or masking, is a strategy to prevent knowledge of the allocated intervention from influencing the behaviour of those involved in the study and thus preventing biases (Kamper, 2018).

In research studies, there are various levels of blinding, and these depend on who has been blinded:

- In unblinded RCTs, participants and/or researchers know who receives which intervention. Unblinded trials are often used when it is not feasible to blind participants or researchers.
- In a single-blind study, usually, the participant is blinded and does not know whether they are in the control or intervention group. However, the researcher or the person delivering the intervention is aware of the group assignments.
- In a double-blind study, both the participant and the individual delivering the intervention are blinded to the group assignments. Usually, in this setup, a third party not involved in the day-to-day operation of the study is responsible for the group assignment.
- In a triple-blind study, this extends to blinding the data analysts in addition to the participants and those delivering the intervention.

In the context of education interventions, blinding can be challenging because the individuals delivering the intervention (teachers, educators, etc.) typically need to know the group assignments to implement the intervention correctly. Similarly, it can be difficult to blind those receiving the intervention (students); for example, they may recognise a different teaching approach. However, when possible, blinding the participants can still help to reduce potential bias if it is possible. For instance, if students are not aware of their group assignments, their performance will be less likely influenced by their knowledge about the intervention. Where it is not possible to blind students or teachers, having assessors blind to the study can be helpful. If blinding was not done, it should be stated that blinding was not done and why.

Example

In this single-blinded study, evaluating a peer tutoring programme, it was possible to blind the outcome assessors to the intervention status of the participants. The marking of the end-of-term assignments, which were the primary outcome measure, was conducted by graduate teaching assistants who were not aware of the student's participation in the peer tutoring programme. This information was kept confidential by the research team, ensuring blinding of the outcome assessment.

- Item 11. Methods (statistical methods): Report (i) statistical methods used to compare groups for primary and secondary outcomes, (ii) methods for additional analyses, such as subgroup analyses and adjusted analyses. State reason if subgroup analyses are not possible or not appropriate.

Providing transparency regarding the analyses allows other researchers to critically evaluate methods and replicate them. The primary and secondary outcomes should clearly be defined. For each of these outcomes, describe the specific statistical tests or models used to analyse the data. This could include t-tests, chi-squared tests, regression analyses, ANOVA, etc., depending on the data type and study objectives. Include any methods used to handle missing data, such as imputation techniques.

Mention the statistical software or programs used to carry out the analyses, including version numbers where relevant. This helps in replicating the study and verifying the results. Discuss any assumptions made by the statistical methods and how these were verified. If sensitivity analyses were conducted to assess the robustness of the findings, describe these as well.

In addition to primary analyses, researchers sometimes conduct additional or secondary analyses to dig deeper into the data. These might include "subgroup analyses", where outcomes are analysed for specific groups within the broader study population, and "adjusted analyses", which account for potential confounding variables. For clarity and replicability, it is important to detail these methods and, if applicable, explain why certain analyses, like subgroup analyses, were not feasible or appropriate.

Example

This study assessed the effectiveness of an innovative teaching strategy on high school students’ mathematics performance (primary outcome) and engagement (secondary outcome). To compare the performance scores between students exposed to the new teaching method (intervention) and those receiving traditional instruction (control), independent t-tests for normally distributed continuous data, and chi-squared tests to analyse differences in engagement levels, categorised as high, medium, or low were employed using SPSS version 26 (IBM Corp.).

Given the multiple comparisons made across different class levels and subjects, a Bonferroni correction to adjust for the increased risk of type I error was applied. Missing data were imputed using the multiple imputation technique.

To ensure the findings' reliability, sensitivity analyses were conducted, examining the effects of different imputation methods on results, thereby assessing the robustness of conclusions against the methods used to handle missing data. The results, including effect sizes and confidence intervals, were presented in a series of tables and figures, illustrating the comparative effectiveness of the new teaching method on student outcomes.

- Item 12. Results (participant flow): For each group, report the numbers of participants (i) who were assigned to or participated in each group, (ii) received intended intervention, (iii) were analysed for the primary outcome, and (iv) (where relevant), report losses and exclusions (after randomisation or group allocation where relevant), together with reasons.

Detailing the participant flow portrays how individuals progress through the study, providing transparency. Balanced attrition across groups avoids biases such as attrition bias, which might arise if there is a differential dropout between groups. Moreover, assessing the response rate helps detect potential non-response bias, where the outcomes might skew if those who respond are significantly different from those who do not. Often this information is communicated via a flow diagram:

- Report the number of participants assigned to each group. Calculate and provide the response rate, representing the percentage of students who completed initial data collection compared to the overall cohort.
- Indicate the number of participants who received the intended intervention.
- Document the number of participants whose data were analysed for the primary outcome. Using this and the initial participation number, derive the attrition rate and the percentage of the initial respondents who remained active until the end.
- Detail losses and exclusions in each group, including reasons for dropout and whether they were similar in both the intervention and control groups (as this may indicate if dropout was related to the intervention).

Example

Out of an initial cohort of 300 psychology students, 250 completed the baseline survey and consented to take part, indicating a response rate of 83.3%. All students assigned to the intervention (*N* = 125) and control group (*N* = 125) received the intended sessions. Out of 250 participants, 220 (88%) completed both the baseline and follow-up surveys. Twenty-five students (20%) from the intervention group dropped out, with 15 (12%) students citing scheduling conflicts and the remaining 10 (8%) indicating dissatisfaction with the tutoring method as their reason for discontinuation. Only 5 (4%) from the control group dropped out, reporting scheduling conflicts.

- Item 13. Results (baseline data): Provide a table showing baseline demographic characteristics for each group.

Presenting baseline demographic data, often summarised in a table, such as age, gender, ethnicity, socio-economic status, or prior educational attainment, aids in understanding the characteristics of study participants. This data should be collected only if it directly aligns with the research aims, objectives, hypotheses, or questions rather than as a routine procedure. When reporting, categorical data (e.g., gender, ethnicity) should be expressed as *N*% while continuous data (e.g., age, prior scores) should be presented using means (M) and standard deviations (SD).

- Item 14. Results (Numbers analysed): Report the numbers analysed for each group (where applicable) and overall cohort.

**Table:** Example Table

	**Intervention ** *N*=50	**Control ** *N*=25
**Age (years), M(SD)**	21 (3.93)	20 (4.78)
**Year of study,** ***N *** **(%)**		
1^st^ year	15 (30)	2 (8)
2^nd^ year	24 (48)	13 (52)
3^rd^ year	11 (22)	10 (40)
**Gender,** ***N*** **(%)**		
Female	46 (92)	23 (92)
Male	2 (4)	2 (8)
Non-binary	1 (2)	-
Prefer not to say	1 (2)	-
**Student status,** ***N*** **(%)**		
Home (UK)	25 (50)	18 (72)
EU	10 (20)	3 (12)
International	15 (30)	4 (16)

This item offers a distinction in reporting study outcomes, focusing on how participants are included in analyses for each outcome. This contrasts with Item 12, which addresses participant dropout and exclusions, detailing who left the study and why. This item, on the other hand, clarifies the number of participants analysed in each group (where relevant) and specifies whether these analyses were based on their original group assignments or on adherence to predefined criteria, such as attending a minimum number of intervention sessions. The latter highlights aspects of intervention fidelity, ensuring the analysis considers only those participants who received a substantial portion of the intervention as intended. By doing so, it allows researchers to assess the effectiveness of the intervention for those who engaged with it adequately, providing a more nuanced understanding of its impact.

Example

In the intention-to-treat analysis (ITT), all students who received the intervention (*n* = 75) and control (*n* = 75) groups were analysed, regardless of their attendance. The per-protocol analysis only analysed participants according to pre-defined engagement criteria, i.e., attending at least 3 out of 5 sessions. For the per-protocol analysis, 60 students in the intervention group met this criterion. The control group did not have session attendance requirements but was analysed for comparative purposes.

- Item 15a. Result (Outcomes and estimation): For each primary and secondary outcome, report (i) the results for each group, at each timepoint. (ii) the estimated effect size and its precision (such as 95% confidence interval)

This item refers to presenting results for each group at every time point. Using tables can make this clearer and more accessible by reporting raw scores; for example, numbers, mean, and standard deviations for each outcome per group. Additionally, the magnitude of the intervention's impact should be illustrated with an estimated effect size, accompanied by its 95% confidence interval for precision. If the study has multiple groups and time points, the statistical analysis should consider both of these aspects. Depending on data distribution, both parametric, like ANOVA with repeated measures, and non-parametric, like the Friedman test, techniques might be relevant.

Example

For math scores, there was a significant main effect of group, F(1, 198) = 24.56, *p* < 0.001, η2 = 0.11, and a significant main effect of time, F(1, 198) = 31.45, *p* < 0.001, η2 = 0.14. The interaction between group and time was also significant, F(1, 198) = 15.67, *p* < 0.001, η2 = 0.07, indicating that the intervention group improved more over time compared to the control group.

**Table:** Comparison of baseline and follow-up scores in reading for intervention and control groups

**Group**	Time	Score, M ± SD
**Intervention**	Baseline	70 ± 5
	Follow-up	85 ± 7
**Control**	Baseline	71 ± 6
	Follow-up	73 ± 6

- Item 15b. Results (outcomes and estimation): for binary outcomes, present both absolute and relative effect sizes

For research involving binary outcomes, absolute and relative effect sizes should be presented. The absolute effect size describes the difference in outcomes between groups in terms of actual risk or probability difference. In contrast, the relative effect size captures the ratio of the likelihood of the outcome occurring in the two groups, helping to contextualise the findings' magnitude and significance. Again, tabulating this information can provide clarity.

Example

Example 1: In a study evaluating the effectiveness of a new reading programme in schools, researchers found that 40% of students in the intervention group passed a literacy test compared to 30% in the control group. The absolute risk difference (effect size) is 10% (40%- 30%). The relative risk (or risk ratio) is 1.33 (40%- 30%), suggesting students in the intervention group were 1.33 times more likely to pass the test.

Example 2: Students in the intervention group (school A) had a higher pass rate on the literacy test compared to the control group (school B). Specifically, 70% of the intervention group (*n* = 70 out of 100) passed the literacy test, whereas 50% of the control group (*n* = 50 out of 100) achieved a passing score. The odds of passing the test for students in the intervention group were 2.33 times that of the control group, OR = 2.33, 95% CI [1.80, 3.00].

- Item 16. Results (ancillary analyses): report the results of any other analyses performed, including subgroup analyses and adjusted analyses

Ancillary analyses refer to additional statistical evaluations not central to the primary study objectives, but which might offer deeper insights into the data. These might include subgroup analyses, where results are stratified by particular subsets of the sample (e.g., by gender or age), or adjusted analyses, which consider extra variables or confounders potentially influencing the outcome. Pre-specified analyses are planned before any data examination or collection, often based on the study's objectives or hypotheses, informed by previous knowledge or assumptions. In contrast, exploratory analyses are undertaken without pre-determined hypotheses and after initial data examination. Their goal is often to discover unexpected patterns, form relationships, or generate novel hypotheses. Differentiating between pre-specified and exploratory analyses reduces the likelihood of practices like 'data dredging', which refers to selectively searching for patterns to support specific conclusions.

Example

In the study assessing a novel teaching approach, a pre-specified subgroup analysis was undertaken to investigate gender-based disparities. An independent-sample t-test revealed significant improvements for both males, t(58) = 2.45, *p* < 0.05, 95% CI [0.25, 1.35], and females, t(58) = 4.30, *p* < 0.001, 95% CI [0.85, 2.15], from pre-to-post intervention. Notably, the effect size was more pronounced for females (d = 0.68, 95% CI [0.56, 0.80]) compared to males (d = 0.49, 95% CI [0.38, 0.60]). A subsequent analysis of covariance (ANCOVA), controlling for socio-economic status, confirmed the significance of the intervention's effect, F(1, 116) = 23.12, *p* < 0.001, η^2 = 0.167*.*

- Item 17. Results (Harms): Report any unintended harms or effects in each group. Indicate how this was handled

While the focus is often on the positive or expected outcomes of an intervention, it is equally important to report any unintended consequences or harms that may arise (Zhao, 2017). This ensures a holistic understanding of the intervention's impact and helps in decision-making for future development and implementation. In the context of educational research, such unintended effects could range from increased stress levels in students to unintended disparities in learning outcomes. Reporting these harms, along with the measures taken in response, enhances the transparency and integrity of the research. If there are no unintended consequences, this can also be stated.

Example

Whilst most students showed improvement in reading abilities, a small subset (6% of the participants) informally and verbally reported increased anxiety and stress related to the heightened workload. This unintended consequence was addressed by modifying their reading tasks to a more manageable pace.

- Item 18. Discussion (Interpretation): State interpretation of results, balancing benefits and harms and considering other relevant evidence.

This section involves a holistic reflection on the study's results, weighing both positive outcomes and potential downsides. It necessitates a balanced appraisal, emphasising not just the beneficial impacts but also any unintended, harmful, or no effects observed. Furthermore, the interpretation should not occur in isolation; it should be grounded in the context of existing literature and evidence. This approach ensures the results are interpreted accurately, promoting credibility and providing readers with a comprehensive perspective on the study's implications and its relevance in the larger academic landscape.

- Item 19. Discussion (Generalisability): Comment on the ability of study findings to be implemented in other contexts.

This section recommends a reflective assessment of the broad applicability of the study's findings. Factors such as having a representative sample might heighten the confidence in generalising results, while a sample predominantly consisting of males or undergraduates might limit the study's applicability to broader populations. For instance, if measures used in the study were replicated at another university, they might be generalisable across similar academic settings. On the other hand, results derived from undergraduates may not necessarily align with postgraduates due to differences in experience, maturity, and academic exposure. Being transparent about the study's context, including individual-level characteristics, assists readers in determining the boundaries within which the study's findings might be relevant. This ensures that insights from the study are used appropriately and are not inadvertently misapplied in unsuitable contexts.

- Item 20. Discussion (limitations): State study limitations.

This section recommends that researchers transparently acknowledge and describe any shortcomings, constraints, or weaknesses of their study. These limitations could arise from the study design, methodology, sample size, or other factors that could potentially influence the interpretation of the findings. This enhances the transparency of the research and provides a context for readers, helping them to critically evaluate the results and implications of the study within its described constraints.

- Item 21. Other information (Registration): State registration number and name of study registry.

The registration of a study in a publicly accessible database is a key part of the research process, enhancing transparency and accountability. This information allows others to access the pre-specified plans for a study, helping to prevent selective reporting, and is often a requirement for many funding bodies and journals.

Example

This study has been registered with the International Standard RCT Number Registry. The registration number is ISRCTN12345678.

- Item 23. Other information (Protocol): Report where the full study protocol can be accessed, if available.

A study protocol is a detailed plan of the study methodology produced before the study is undertaken. It enhances transparency and replicability. If a protocol exists, its availability should be stated, including details about where it can be accessed.

Example

The complete protocol for this study is available on the Open Science Framework (OSF). It can be accessed at the following URL: [enter URL].

- Item 24. Other information (Funding): State the sources of funding and other support, the role of funders.

Stating the funding source allows readers to assess potential conflicts of interest and understand the role of the funding bodies in the research process, if any. Even if there was no direct funding for the research, any indirect support, such as the provision of resources, should be acknowledged. It could also increase research credibility, as it shows a level of additional checking and evaluation.

Example

This research was funded by the National Institute of Education (grant number NIE-EDU-2022–4567). The funder had no role in the study design, data collection, data analysis, interpretation, or writing of the report. Administrative and logistical support was provided by [NAME University's Department of Education].

- Item 25. Other information (data repository): Report the use of any data repository.

Sharing research data in the public domain allows for independent verification of results, and facilitates further research by others. If data from the study have been or will be, shared in a data repository, this should be stated, including the name of the repository and any access restrictions. If data are not shared, reasons should be given, such as privacy concerns or specific research grant terms.

Example

The anonymised data from this study have been deposited in the [NAME] University Data Repository. These data can be accessed upon request, adhering to data protection regulations.

- The CIDER checklist explanation and elaboration.

As previously stated, the CIDER checklist fits into Sect. 5a of the CLOSER checklist. The CIDER checklist guides researchers in comprehensively detailing educational interventions (and control conditions). Should word count constraints arise, supplementary data or external references should be utilised to provide a full description. This thorough reporting ensures replicability, helps determine effectiveness, and highlights areas for refinement in education interventions.

Similarly to our elaboration on the CLOSER checklist, we provide an explanation and elaboration for each item on the CIDER checklist, supported by relevant examples. As previously stated, the text for each example may not directly translate to every scenario and should be adapted based on the specific context of the intervention. Some items might not be mutually exclusive, and certain aspects of the intervention might be better articulated by combining specific items. The order of the items serves as a guideline only.

- Item 1. Brief title of intervention: provide the name or a phrase that captures the key 'essence' or intention of the intervention.

A succinct title encapsulating the essence of the intervention helps readers quickly grasp its focus. A well-chosen title not only informs but also aids in differentiating the intervention from similar initiatives. It should be representative of the content, target group, desired outcome of the intervention, and study design, ensuring clarity and reducing ambiguity.

Example

Numeracy Boost: A Randomised Control Trial of a Tailored Mathematics Enrichment Programme Supporting Numeracy Skills for Children aged 6–8 years.

- Item 2. Aim and objectives of intervention: succinctly state: (i) overall aim of the intervention; (ii) objective(s) and/or intended learning outcome(s) of the intervention.

By providing both aims and objectives, researchers and educators can align their activities and evaluations to these predefined goals, gauging the intervention's success relative to its intended purpose. The aim presents a broad overview of the intervention's primary goal, while the objectives and/or intended learning outcomes offer a detailed breakdown of what the intervention seeks to achieve on a more specific level.

Example

The programme aimed to promote mental wellbeing among undergraduate students through mindfulness practises. Its specific objectives were to (i) introduce students to mindfulness techniques tailored to academic stressors, and (ii) provide strategies for integrating daily mindfulness practices into their routines. By the end of the program, students were expected to be confident in utilising mindfulness exercises to reduce anxiety, enhance focus, and improve their overall emotional resilience during demanding academic periods.

- Item 3. Rationale for intervention: report the purpose of intervention development with reference to relevant theory or observations

This item might be positioned in the introduction or methods section of a manuscript. The rationale establishes the intervention’s foundation, anchoring it in academic theory, research, or observed phenomena. For example, a clear presentation of the theoretical, empirical (e.g., surveys, interviews, or focus groups), or observational (e.g., classroom observations or insights from student-facing roles such as counselling services or Students' Union) background ensures the intervention's relevance and potential impact are transparent. This section might also elaborate on prior interventions that influenced the current one's formulation, either by building on the strengths of existing strategies or critiquing them to justify the need for a new intervention in a specific context.

Example

The workshop was developed following observed polarisation among politics students during classroom debates. Drawing from the theories of deliberative democracy, the intervention was designed to foster open dialogue and encourage empathetic listening. This theory-based approach was reinforced by academic observations pointing to the enhanced learning experiences when students engage in constructive, rather than divisive, political discussions.

- Item 4. Participants: (i) Report who the intervention was developed for. (ii) Report if the intervention was open to all participants across the institution or for a specific cohort. (iii) Report any collected participant demographic data such as age, gender ethnicity, socioeconomic status.

Delineating the target audience of the intervention and specifying whether the intervention is open to all students or targeted towards a specific population, aids in understanding its context, scope and intention. This can be supported with an in-text description and a table outlining demographic data (including protected characteristics where relevant to the research hypotheses) using appropriate statistical measures, such as mean and standard deviation for continuous variables or frequency and percentage for categorical ones, ensuring a comprehensive portrayal of the participant profile.

Example

The programme was tailored for international students entering their first year of university. Though the course was initially curated for students facing language barriers, it was subsequently opened to any interested international student. Fifty-five students took part in the programme. The average age of participants was 20 (SD = 2.4), 55% identified as male, and the majority of the sample was Asian ethnicity (50%) (see Table XX).

- Item 5a. Intervention provider(s): (i) Indicate who provided the intervention, e.g., state the job title/role of instructor(s) and the education organisation providing the intervention. (ii) Report the relationship between intervention provider and developer, (iii) Comment on any prior relationship between participants and instructors. (iv) Report the instructors’ expertise, professional discipline, background and any relevant training they have undertaken.

Detailing intervention providers and capturing the credentials and affiliations provides insight into potential influences that might affect the programme's delivery. Clearly indicating whether providers are internal or external to the department or institution offers a context about their proximity to the target audience. The nature of the relationship between students and instructors, whether they are personal tutors, module leaders, peers, or department heads, can have implications for the intervention's efficacy. For clarity:

- Providers refer to the hosting education organisation or an external body.
- Developers are those who conceptualised and designed the intervention.
- Instructors are the individuals who directly delivered the intervention to the students (the developer and instructor might be the same person).

Example

The workshop was provided within the Sociology Department of a large urban university in the UK and was developed by Inclusivity Now, an external organisation, in tandem with university curriculum developers. It was delivered by a Teaching Assistant from the Sociology Department. Some participants had previously interacted with the instructor as he was also a module leader for certain courses. The instructor has a Master's in Sociology and training in inclusive teaching methods and was selected as the instructor to bridge the content created by Inclusivity Now with the students' curriculum experiences.

- Item 5b. Intervention developer(s): (i) Report who designed the intervention (developer[s]) and their professional background(s) and expertise. (ii) Report whether developer(s) were also involved in delivering the intervention (instructor[s]) and/or trained the instructors. (iii) Report whether participants (particularly those with lived experience where relevant) were involved in co-creation of the intervention. If so, explain their involvement, if not, provide reasoning.

Knowing who developed the intervention offers insights into the expertise and perspectives that shaped it. Detailing the professional backgrounds of the developer(s) can lend credibility and indicate the extent of domain-specific expertise. Moreover, understanding if developers were also instructors can inform potential biases or unique insights in the study results. Similarly, detailing the involvement of participants (could include those with lived experiences where relevant), indicates the intervention's responsiveness to real-world needs and the potential for enhanced relevance and effectiveness.

Example

This module (Cultural Anthropology) was designed by two primary developers (JS and RM) who have a PhD in Cultural Anthropology with 15 years of fieldwork experience in sub-Saharan Africa, and a PhD in Sociology specialising in rural communities, respectively. JS was the primary instructor for the course and RM was responsible for training two adjunct instructors, teaching assistants in the Sociology Department, in the course methodology and content. To ensure the intervention's relevance and breadth, three students from the previous year's cohort, all international or first-generation college students, were actively involved in refining the curriculum for the module. This collaborative approach aimed to infuse diverse cultural perspectives into the course content.

- Item 6a. Institutional setting: Indicate whether the intervention was conducted across one or multiple institutions. For each institution, report their nature and location, e.g., the nature of the institution (i) Urban or rural; (ii) School, further education, higher education; (iii) Teaching on campus, online or both. Report (iv) the size of the institution, i.e., total student population.

The institutional details, such as its geographical location (urban or rural) and the mode of teaching, can influence intervention delivery and reception. Further, knowing whether the intervention was carried out in one or multiple institutions gives insight into its adaptability across different environments. Reporting the size of the institution, or the total student population, can also help gauge the scale at which the intervention operated. This could be stratified by type of student to add depth, for instance, outlining the total number of full-time undergraduate students if they were the primary target. By discussing the institutional setting and hence shedding light on the cultural backdrop, readers are better positioned to understand any cultural nuances or considerations that may have affected the intervention's outcomes. Additional aspects like the precise geographical location of the institution and the primary language(s) spoken can further enrich this context. However, care must be exercised when revealing specific geographical details to ensure participant anonymity remains uncompromised.

Example

The intervention was rolled out across two institutions, where all teaching was performed in-person to pupils aged 11–16. The first was a large urban secondary school located in central Birmingham with a total student population of 1,350. The school, with its diverse multicultural student body, added a rich cultural dimension to the intervention. The second institution was a medium-sized mixed urban–rural secondary school situated on the outskirts of Leeds, catering to around 950 students.

- Item 6b. Intervention location: (i) Report the location(s) where the intervention occurred, e.g., classroom, lecture theatre, online, workplace placement, off-campus visits, green space. (ii) Include relevant infrastructure details, e.g., size of space, formal or informal layout.

By detailing the exact spaces where interventions took place, such as classrooms, lecture theatres, or even outdoor areas, researchers and readers can gain insight into the environmental factors that might have played a role in the study's outcomes. Furthermore, infrastructure details, like the size of the space or the layout's formality, can provide context regarding the settings' conduciveness for learning or the kinds of interactions it might promote.

Example

The intervention predominantly took place in the college's main art studio, a spacious, naturally lit room that accommodates up to 30 students. The room's informal layout, with movable worktables and an open floor plan, was designed to promote collaboration and free movement.

- Item 7. Intervention timing and duration: Indicate (i) The number of times the intervention was delivered, and over what period of time. (ii) The number of sessions (and any home practice where relevant). (iii) If the content was self-paced, how long the content was designed to be completed in (e.g., 30 h across two semesters), and what guidance was given to the participants. (v) The time of year/semester the intervention was delivered, including any other time-appropriate information, e.g., delivered close to the exam period.

Detailing when the intervention was conducted, how many sessions it encompassed, and its overall length helps provide clarity about the commitment required from participants. This information also allows for a clearer assessment of the feasibility of the intervention in other settings. Factors such as the time of year or semester can influence students' reception and engagement, given other academic or personal commitments. For interventions that are self-paced, it is essential to note the expected duration for content completion, which offers a sense of the intervention's depth and breadth. Stating how many terms or semesters were in the academic year will also provide context.

Example

The intervention consisted of eight weekly sessions spanning two months, delivered in the first term from mid-October (academic year comprised of two terms). Each session lasted 90 min, and students were also provided with weekly home practice assignments, expected to take roughly 30 min each. Although the content was not entirely self-paced, the last two sessions were designed as 'flexible labs' where students could explore topics of interest, spending three hours over those two weeks, with guidance on potential areas of focus provided. Notably, the intervention concluded three weeks before the end-of-term exams, providing students with time to complete pre-exam revision.

- Item 8a. Delivery – Modes and content: (i) Briefly outline main delivery method(s) for the intervention, e.g., face-to-face lectures, seminars, workshops; online videos, discussion. (ii) State whether activities were individual or group-based; instructor-led or self-paced by participants. (iii) Provide an outline of main content for each session or activity. (iv) Report the ratio of participants to instructors.

Stipulating whether sessions were held face-to-face or online, the nature of activities (individual or group-based), and the style of leadership (instructor-led, student-led, or self-paced) provides readers with information about the teaching environment and how the intervention was executed. Specifically, if activities were student-led, it is helpful to report what support was extended by staff to facilitate this pedagogical approach. Outlining the primary content for each session or activity offers clarity regarding the topics covered, progression, and depth of learning. Reporting the ratio of participants to instructors offers insights into the level of personalised attention, interaction, and feedback that participants might have received. Moreover, making the content publicly accessible is highly encouraged. This not only boosts the transparency of the research but also aids in replication, further development, and adaptations by others in different educational, social or cultural contexts.

Example

The main delivery methods were face-to-face seminars, coupled with pre-seminar online video materials and discussion forums. While seminars were instructor-led, focusing on critical readings and analysis of chosen texts, online video sessions allowed students to explore the historical and societal contexts at their own pace. Activities within the seminar were a mix of individual reflections and group discussions. Over 12 weeks, the course covered iconic modernist authors, with each week dedicated to a specific author or thematic topic (see supplementary material for more detailed information). With 30 students enrolled and two instructors facilitating, the student-to-instructor ratio was 15:1.

- Item 8b. Delivery – Procedures: Indicate whether the intervention was (i) Embedded in the core curriculum, e.g., formally taught, included in assessment design, aligned with pedagogical approach; or (ii) Run outside of the core curriculum. If the intervention was curriculum embedded, indicate whether the intervention was credited, compulsory, and/or assessed (add details of assessment if relevant). Report the nature of any ‘home practice’ participants were given. (ii) Report any incentives offered to student participants.

Specifying whether the intervention was embedded within the core curriculum or operated externally is vital to understanding its integration and importance within the educational framework. For example, if the intervention is delivered within the core curriculum, it is typically perceived as central to the student's educational journey, potentially influencing participation rates and overall engagement. Factors like credit assignment, compulsory nature, and modes of assessment further shape this landscape, often determining the gravity and commitment students allocate to the intervention. The methods of assessment (e.g., written exams, oral presentations, project submissions, or practical evaluations) play a role in shaping students' approach to the intervention. Contrastingly, interventions operating outside the core curriculum might need additional motivators, like incentives, to drive participation. Reporting any home practice elements gives insight into the expected individual efforts beyond the structured learning environment. Details about incentives, if provided, add transparency regarding external motivating factors influencing student participation.

Example

The intervention was embedded within the core curriculum. The student project was credited and compulsory, contributing to 20% of the student's final grade. Students were required to deliver a group presentation assessed by a panel of three teachers. Outside of classroom sessions, students were required to engage in home practice, documenting their personal efforts to reduce waste and increase recycling in their households over four weeks. While motivation was expected, given the intervention's curriculum weightage, participating students were also incentivised with the opportunity to present their findings at the county's annual sustainability conference, a platform for student voices.

- Item 9. Materials: Report any physical or informational materials used in the intervention, including those (i) provided to participants; (ii) used in intervention delivery; (iii) used in training of intervention instructors. Indicate where the materials can be accessed.

Detailing the materials allows for transparency, reproducibility, and potential adaptation by other educators or researchers in varied contexts. Physical materials could range from tangible tools to worksheets, while informational materials might encompass readings, multimedia content, or online resources. If the intervention involved special training for instructors (including student instructors if relevant), any resources or manuals employed during that training is also useful detail. Making these materials publicly accessible enhances the broader educational community's capacity to evaluate, replicate, or adapt the intervention, promoting a more open and collaborative educational research environment.

Example

Materials included: (i) For participants, a curated workbook with images of artefacts, related readings, and reflection spaces, and a "Viking Artefact Kit" with replicas of Viking tools and coins; (ii) For intervention delivery, a digital slideshow comparing artefacts with modern items, an interactive digital quiz on artefacts, and short documentary videos about Viking daily life; (iii) For instructor training, a detailed instructor's guidebook, and online seminars by Viking-era historians. All materials are available on the school’s “History Through Artefacts” webpage, licensed under creative commons, for educators wishing to adapt or replicate the programme.

- Item 10a. Evaluation – Attendance: Indicate (i) The number of participants who participated in the intervention. If the intervention included multiple sessions, indicate participant numbers for each one, and total of participants who completed the whole intervention, or dropped out mid-way. (ii) How attendance figures were recorded, and by whom. (iii) Any strategies that were used to facilitate attendance

Recording who attended, how often they engaged, and how many completed or dropped out offers insight into the intervention's feasibility and acceptability. Further, it can help understand patterns or factors affecting participation, which could be crucial for refining or scaling the intervention. Detailing the methods employed to capture attendance and any strategies deployed to boost it not only aids in a critical evaluation by other researchers but also facilitates replication. In certain instances, specifying the number of sessions necessary to reach the intervention's threshold (compliance) is appropriate. This will influence subsequent analyses, for instance, evaluating those who met the minimum intervention requirements.

Example

A total of 150 students enrolled in the course, which was structured into 10 weekly sessions. On average, 140 students attended each session, with 130 students completing the entire course and ten dropping out after the fifth session. Students had to attend nine of the ten sessions to be recorded as having completed the course. Attendance was automatically recorded by the online learning platform, and a teaching assistant manually cross-checked the list for any discrepancies or technical glitches. To facilitate consistent attendance, students were given access to supplementary resources after each session and were incentivised with a certificate of completion at the end of the course. Furthermore, reminder emails were sent out 24 h and one hour before each session.

- Item 10b. Evaluation – Delivery: (i) Report any form of observation or assessment of the intervention delivery, i.e., instructor observation, student satisfaction questionnaire.

Monitoring how the content and methods are executed in practice can reveal discrepancies between the planned and actual intervention delivery. Various mechanisms can be employed to assess this, from instructor observations to student feedback via questionnaires. This feedback helps identify areas of improvement, ensures that the intervention was delivered as intended, and captures the participant's perspective on the content and methods used. Evaluating delivery also considers the instructor's proficiency, adherence to the intervention protocol, and the overall student reception of the intervention.

Example

To evaluate the delivery of this programme, two primary methods were utilised: firstly, external educators were brought in to observe the lessons and note any deviations from the planned lesson structure, and any particularly effective methods or strategies used by the instructor. Secondly, at the conclusion of the intervention, students were provided with a satisfaction questionnaire, asking them to rate the clarity of the instruction, the usefulness of the materials provided, and their overall satisfaction with the programme. The combination of observational and feedback methods offered a comprehensive view of how the intervention was delivered and received.

- Item 10c. Evaluation – Intervention design: Indicate the extent to which the intervention was delivered as planned with regard to: (i) Participants, e.g., whether they constituted a representative demographic sample of the institution’s student cohort. (ii) Intervention materials. (iii) Procedures. (iv) Instructors. (v) Mode of delivery. (vi) Location and setting. (vii) Number of sessions and their frequency. (viii) Timing and duration of the intervention

By examining components such as the participants, the materials used, the procedures followed, the instructors' expertise, the mode and location of delivery, session details, and the overall timing and duration, researchers can discern if any deviations from the planned intervention might have influenced the outcomes. An essential aspect to consider is time and workload constraints, which can significantly affect the ability to deliver the intervention as initially intended. These practical challenges might necessitate some tailoring or adjustments. Importantly, it is recognised that tailoring an intervention to the specific needs or constraints of a context is likely and even desirable in many cases. What is critical is transparency in reporting any such changes, along with the reasons for them. The aim here is not to judge 'how well' an intervention was delivered, but rather to understand its context, the decisions made, and any adaptations, ensuring a comprehensive view of the intervention's implementation.

Example

On evaluation, it was found that the participation mainly involved second-year students, even though it was aimed at all year groups. While all the designated e-books and videos were provided to participants as planned, there was an unanticipated change in one instructor due to unforeseen commitments. The course remained entirely online, utilising the college's learning management system. The setting was consistent, with all sessions taking place in virtual classrooms. The programme was comprised of eight weekly sessions, each lasting 90 min, in alignment with the initial design. However, due to the intervention's timing coinciding with peak assignment periods, some content delivery was modified to accommodate students' workload, a change that was transparently communicated to ensure the primary objectives of the workshop were met without overwhelming the students.

- Item 10d. Evaluation – outcome measurements: indicate whether the outcome of the intervention was directly measured, e.g., in a mindfulness intervention, was mindfulness measured pre- and post-intervention.

Whilst it is crucial to measure any primary and secondary outcomes aligned with the study hypotheses, it is also helpful to check the intervention occurs as expected for several reasons. Firstly, it ensures that the intervention is being assessed on its intended purpose and not on peripheral or unintended benefits in order to discern the direct effectiveness of the intervention. Secondly, a well-aligned outcome measurement offers clarity to stakeholders, making it evident what the intervention sets out to achieve and what change can be expected. Thirdly, by measuring outcomes directly related to the intervention’s aim, it becomes feasible to refine the programme in subsequent iterations, focusing on enhancing its strengths and rectifying its limitations. Lastly, such an approach facilitates comparison with other similar interventions, creating a benchmark for best practices. Thus, when evaluating interventions like physical activity or mindfulness, it is imperative to measure physical exertion and mindfulness, respectively, alongside any outcome measures related to the hypothesis, e.g., study stress.

Example

In a secondary school setting, a mindfulness meditation programme was introduced to help students aged 14–15 manage exam-related anxiety. The primary outcome measure of the study was exam-related anxiety which was assessed using the State-Trait Anxiety Inventory (STAI) questionnaire. Alongside this measure, to ensure that the programme did impact mindfulness practice, the 15 items Mindful Attention Awareness Scale (MAAS) was used to measure mindfulness pre- and post-completion of the mindfulness medication programme. The STAI and MAAS scores were collected twice: once before the commencement of the eight-week meditation programme and once after its conclusion.

- Item 11. Modifications to intervention: If the intervention design was modified during the study, indicate the changes (what, why, when, and how) and the reasons for the changes.

Detailing modifications made to an intervention (what, why, when, and how) during its course allows researchers, educators, and stakeholders to understand the intervention's adaptability and responsiveness to unforeseen challenges or new insights, enhancing transparency and integrity. Furthermore, it offers a window into the decision-making processes, allowing for more nuanced interpretations of the results and providing insights for those considering similar interventions, and implementation of real-world interventions.

Example

Initially, online video lectures were assigned for home viewing, and in-class sessions were dedicated to problem-solving workshops. However, midway through the semester, feedback indicated that students felt overwhelmed with the length of video lectures. Thus, the intervention was modified. The video lectures were broken down into shorter, more digestible segments, and supplementary reading materials were introduced to provide additional context. Supplementary material was provided 1 week in advance and in an electronic format (such that could be enlarged or accessed via a screen reader). This change, made in week 6 of a 12-week semester, aimed to make the pre-class preparation more manageable, effective and inclusive for all students, including those with a disability. The decision to modify was driven by ongoing formative feedback from students and the course instructor's observations.

## Discussion

Inadequate reporting of educational interventions hinders progress in research by limiting replicability and implementation. Studies often lack clear, thorough descriptions of interventions, with key information frequently missing (Abulfaraj et al., 2024; Meinema et al., 2019; Upsher et al., 2022). A review of evidence-based practice educational interventions emphasised the importance of comprehensive reporting for translating research into practice (Albarqouni et al., 2018).

We identified a significant gap in the tools available for comprehensive reporting of diverse methodologies in educational intervention research. To fill this gap, we developed tailored checklists grounded in expert consensus, designed specifically for manuscript preparation across various quantitative study designs and educational contexts. The importance of such domain-specific tools cannot be overstated, as they can ensure more transparent and thorough research reporting (Brand, Hardy, & Monroe, 2020; Kousta et al., 2020). By promoting detailed and adaptable reporting practices, these checklists can enhance both the consistency and accessibility of research findings, aligning with recent calls for increased transparency in educational research (Ziegler et al., 2021).

Importantly, the expertise of the research team and participants of the Delphi consensus survey spans both quantitative and qualitative research domains, enabling us to bridge traditional research paradigms and fields such as education and health. This interdisciplinary approach not only enhances the utility of our checklists but also promotes accessible and transparent reporting that transcends paradigmatic and disciplinary boundaries. The checklist feedback underscores the collaborative spirit of this work, aiming to enhance connectivity across diverse research methodologies within educational research. This approach facilitates a deeper, shared understanding among researchers, regardless of their primary methodological orientation. Such clarity and detail in reporting are foundational for enriching scholarly discussions and advancing collective knowledge in educational research.

The checklists advocate for evidence-informed rather than merely evidence-based practice (De Bruyckere, 2018). They emphasise the importance of clear reporting that allows educators to tailor interventions to their specific contexts and subsequently report on their efficacy. This approach aligns with the broader educational mandate of fostering adaptable and contextually relevant teaching and research practices.

Furthermore, it is essential to acknowledge that while our focus has been on enhancing the reporting of quantitative research, the principles embedded in our checklists, particularly the CIDER checklist, are equally applicable to qualitative methods sections. This does not imply an endorsement of any specific research design; rather, it reflects the recognition of the complementary value of both qualitative and quantitative approaches in evaluating educational interventions for a holistic understanding of educational phenomena.

### Limitations

Despite rigorous development, the CLOSER and CIDER checklists have limitations. First, while the items provide detailed reporting guidelines, some educational interventions, especially in culturally unique contexts, may require further adaptation. Moreover, the consensus achieved through Delphi rounds, while extensive, may reflect the perspectives of a specific academic sample, warranting further validation across broader educational disciplines. Although contributors worked in a range of countries, the majority were based in the UK, Australia and the US, meaning other regions were less well-represented. As such, further validation may be needed, such as validating the checklists in non-English-speaking countries could promote a more balanced representation of participants from diverse linguistic and cultural backgrounds. Educational intervention research often fails to report and analyse race and ethnicity adequately, with many studies underrepresenting minority groups and neglecting to examine potential impacts on reducing racial/ethnic disparities (Gaias et al., 2020). Improving the completeness and uniformity of intervention descriptions, including participant demographics, is crucial for advancing educational research and effectively addressing educational disparities. Awareness of CLOSER and CIDER checklists when designing interventions may also help reduce these disparities.

Given the inherent diversity of educational settings—spanning macro (systemic), meso (institutional), and micro (individual) levels, adherence to standardised criteria may sometimes challenge the specificities of each context. Therefore, while these checklists offer a structured framework, they are designed with flexibility in mind, allowing researchers to adapt items as needed to account for unique aspects of their research environments. This adaptability is essential in balancing robust evaluation with practical applicability, ensuring that researchers can maintain transparency and rigour while respecting the nuances of their specific settings.

### Future directions

A crucial next step is to evaluate these checklists' applicability and effectiveness in academic settings. Drawing inspiration from checklists in health research, our post-publication strategy for these educational checklists includes promoting their endorsement, aiding researchers in their application, and assessing their overall impact (Moher et al., 2010). However, it is important to recognise that checklists by themselves cannot elevate reporting standards in education intervention research (Phillips et al., 2016). Hence, there is a pressing need for educational institutions, journals, and researchers to adopt and champion these tools (Ryan et al., 2023). Doing so ensures consistent and replicable reporting across the spectrum of quantitative study designs in education intervention research (Anderson et al., 2021).

### Conclusion

In conclusion, the CLOSER and CIDER checklists provide foundational tools aimed at improving reporting standards in educational intervention research. These checklists address a critical gap in standardised criteria for describing quantitative educational interventions, supporting researchers in producing transparent and replicable reports. Their flexible structure allows for adaptability across varied educational contexts, enhancing both the evaluation and application of research findings. By enabling clear communication of study methods and results, CLOSER and CIDER contribute meaningfully to the dissemination and implementation of evidence-based practices in education, advancing the field toward a more rigorous and impactful future. Future research should explore the application of these checklists across diverse educational settings to establish their robustness and further refine their scope.

## Supplementary Information

Supplementary Material 1.

## Data Availability

All data generated or analysed during this study are included in this published article and its supplementary information files.
