# Supplementary material for: Improving reporting standards in quantitative educational intervention research: introducing the CLOSER and CIDER checklists
Source: J New Approaches Educ Res. 2025 Feb 3;14(1):2. doi: 10.1007/s44322-024-00022-9 (PMC11801185; doi:10.1007/s44322-024-00022-9)
Supplement: Supplementary file 1 — Supplementary Material 1. [file 44322_2024_22_MOESM1_ESM.docx]

# Supplementary Data

## Supplementary S1: First drafts of the CLOSER and CIDER checklists following Stage 1 (ready for Stage 2 expert team meeting)

| The first draft of the CLOSER checklist | | | | |
| --- | --- | --- | --- | --- |
| Section/Topic | Item No | Checklist item from CONSORT | Checklist item adapted for CLOSER | Notes on adaptation |
| Title and abstract | | |  |  |
|  | 1a | Identification as a randomised trial in the title | State study design (RCT, non-RCT, pre-post without control) in the title | Expanded for inclusion of multiple study designs |
|  | 1b | Structured summary of trial design, methods, results, and conclusions (for specific guidance see CONSORT for abstracts) | Structured summary of design, methods, results, and conclusions in the abstract | Deleting word ‘trial’ for inclusion of other study designs |
| Introduction | | |  |  |
| Background and objectives | 2a | Scientific background and explanation of rationale | Theoretical and educational (and other context relevant) background and explanation of rationale | Terminology adapted for use in education context |
|  | 2b | Specific objectives or hypotheses | Specific objectives or hypotheses | No change |
| Methods | | |  |  |
| Study design | 3a | Description of trial design (such as parallel, factorial) including allocation ratio | Description of study design (such as RCT, non-RCT, or pre-post without control) | Expanded for inclusion of multiple study designs. |
|  | 3b | Important changes to methods after trial commencement (such as eligibility criteria), with reasons | State any changes to methods after study started (such as eligibility criteria), with reasons | Terminology adapted for use in education context |
| Participants | 4a | Eligibility criteria for participants | Eligibility criteria for participants | No change |
|  | 4b | Settings and locations where the data were collected | Settings and locations where the data were collected | No change |
|  | 4c | Dates defining the periods of recruitment and follow-up | Dates defining the periods of recruitment and follow-up | No change (this item was in results section in consort 2010) |
|  | 4d | Why the trial ended or was stopped | Why the study ended or was stopped | Changing ‘trial’ to ‘study’ to acknowledge other study designs (this item was in results section in consort 2010) |
| Interventions | 5 | The interventions for each group with sufficient details to allow replication, including how and when they were actually administered | Details of intervention (and control if applicable) with sufficient details to allow replication. Please follow CIDER checklist. | Terminology adapted for use in this context. Advises uses of CIDER checklist. |
| Outcomes | 6a | Completely defined pre-specified primary and secondary outcome measures, including how and when they were assessed | Completely defined pre-specified primary and secondary outcome measures, including how and when they were assessed | No change |
|  | 6b | Any changes to trial outcomes after the trial commenced, with reasons | Any changes to outcomes after the study commenced, with reasons | Deleting word ‘trial’ for inclusion of other study designs |
| Sample size | 7a | How sample size was determined | How sample size was determined | No change |
|  | 7b | When applicable, explanation of any interim analyses and stopping guidelines | When applicable, explanation of any interim analyses and stopping guidelines | No change |
| Randomisation and bias: |  |  |  |  |
| **For non-RCTs and pre-post without controls, complete section 8aa, and then skip to 12a.**  **For RCTs, skip to 8ab** | | | | |
| Bias | 8aa | Bias | Identify potential sources of bias. Describe any efforts to address potential sources of bias | Added from STROBE checklist to account for other study designs |
| Sequence generation | 8ab | Method used to generate the random allocation sequence | Method used to generate the random allocation sequence | No change |
|  | 8b | Type of randomisation; details of any restriction (such as blocking and block size) | Type of randomisation (e.g., computer generated, coin flip, quasi random etc); details of any restriction (such as blocking and block size) | Examples added for clarification |
| Allocation concealment mechanism | 9 | Mechanism used to implement the random allocation sequence (such as sequentially numbered containers), describing any steps taken to conceal the sequence until interventions were assigned | Mechanism used to implement the random allocation sequence (such as sequentially numbered containers), describing any steps taken to conceal the sequence until interventions were assigned | No change |
| Implementation | 10 | Who generated the random allocation sequence, who enrolled participants, and who assigned participants to interventions | Who generated the random allocation sequence, who enrolled participants, and who assigned participants to interventions | No change |
| Blinding | 11a | If done, who was blinded after assignment to interventions (for example, participants, care providers, those assessing outcomes) and how | Who generated the random allocation sequence, who enrolled participants, and who assigned participants to interventions | No change |
|  | 11b | If relevant, description of the similarity of interventions | If relevant, description of the similarity of interventions | No change |
| Statistical methods | 12a | Statistical methods used to compare groups for primary and secondary outcomes | Statistical methods used to compare groups for primary and secondary outcomes | No change |
|  | 12b | Methods for additional analyses, such as subgroup analyses and adjusted analyses | Methods for additional analyses, such as subgroup analyses and adjusted analyses. State reason if subgroup analyses are not possible or not appropriate. | Adding in “State reason for subgroup analyses not possible or not appropriate.” to acknowledge potential RoB criteria. |
| Results | | |  |  |
| Participant flow (a diagram is strongly recommended) | 13a | For each group, the numbers of participants who were randomly assigned, received intended treatment, and were analysed for the primary outcome | For each group, the numbers of participants who were assigned to each group, received intended intervention, and were analysed for the primary outcome | Terminology adapted for use in education context and for multiple study designs |
|  | 13b | For each group, losses and exclusions after randomisation, together with reasons | For RCTs: For each group, losses and exclusions after randomisation, together with reasons  For non-RCTs: For each group state reasons for exclusions after group allocations, and reasons for loss to follow-up.  Pre-post without controls: N/A | Adding ‘For RCTs’  Adding sentence for non-RCTs |
| Baseline data | 14 | A table showing baseline demographic and clinical characteristics for each group | A table showing baseline demographic and demographic characteristics for each group | Terminology adapted for use in education context |
| Numbers analysed | 15 | For each group, number of participants (denominator) included in each analysis and whether the analysis was by original assigned groups | For each group (where applicable), number of participants (denominator) included in each analysis and whether the analysis was by original assigned groups | Terminology adapted for use in multiple study designs |
| Outcomes and estimation | 16a | For each primary and secondary outcome, results for each group, and the estimated effect size and its precision (such as 95% confidence interval) | For each primary and secondary outcome, results for each group and timepoints. For example, at baseline and follow-up, and for each group, report the sample size, mean and standard deviation. Report p-values for differences between groups and timepoints, the estimated effect sizes and its precision (such as 95% confidence interval).  A table to represent this information is encouraged.  If there are 2 groups and 2 timepoints, an analysis that takes into consideration both time and group should be reported. | Examples of type of data that should be reported is given. Tables and time by group analyses are encouraged. |
|  | 16b | For binary outcomes, presentation of both absolute and relative effect sizes is recommended | For binary outcomes, presentation of both absolute and relative effect sizes is recommended | No change |
| Ancillary analyses | 17 | Results of any other analyses performed, including subgroup analyses and adjusted analyses, distinguishing pre-specified from exploratory | Results of any other analyses performed, including subgroup analyses and adjusted analyses, distinguishing pre-specified from exploratory | No change |
| Harms | 18 | All important harms or unintended effects in each group (for specific guidance see CONSORT for harms) | All important harms or unintended effects in each group | No change |
| Discussion | | |  |  |
| Limitations | 19 | Trial limitations, addressing sources of potential bias, imprecision, and, if relevant, multiplicity of analyses | Study limitations, addressing sources of potential bias, imprecision, and, if relevant, multiplicity of analyses | Changing ‘trial’ to ‘study’ to acknowledge other study designs |
| Generalisability | 20 | Generalisability (external validity, applicability) of the trial findings | Generalisability (external validity, applicability) of the study findings | Changing ‘trial’ to ‘study’ to acknowledge other study designs |
| Interpretation | 21 | Interpretation consistent with results, balancing benefits and harms, and considering other relevant evidence | Interpretation consistent with results, balancing benefits and harms, and considering other relevant evidence | No change |
| Other information | | |  |  |
| Registration | 22 | Registration number and name of trial registry | Registration number and name of study registry | Changing ‘trial’ to ‘study’ to acknowledge other study designs |
| Protocol | 23 | Where the full trial protocol can be accessed, if available | Where the full study protocol can be accessed, if available | Changing ‘trial’ to ‘study’ to acknowledge other study designs |
| Funding | 24 | Sources of funding and other support (such as supply of drugs), role of funders | Sources of funding and other support, role of funders | Terminology adapted for use in education context |

| **The first draft of the CIDER checklist** | | | |
| --- | --- | --- | --- |
|  | **Checklist item from TIDieR** | **Checklist item adapted for CIDER** | **Notes regarding adaption** |
| **1** | Brief name  Provide the name or a phrase that describes the intervention | Brief name  Provide the name or a phrase that clearly describes the intervention | No change |
| **2** | Why  Describe any rationale, theory, or goal of the elements essential to the intervention | Why  Describe why the intervention was developed with any reference to theory or previous interventions that aided intervention development. | Terminology adapted for education context |
| **3** | TailoringIf the intervention was planned to be personalised, titrated or adapted, then describe what, why, when, and how | Who ForDescribe who the intervention was developed for (subject cohort and level of study) and who could access it (open to all or targeted population of students e.g. students with anxiety, female students, students struggling with a component of the curriculum) | This subsection has moved from item 9 in the original TIDieR checklist to item 3 here to reflect importance. The title has been changed from ‘tailoring’ to ‘who for’ to encourage detailed reporting of the target population |
| **4** | What (materials)Describe any physical or informational materials used in the intervention, including those provided to participants or used in intervention delivery or in training of intervention providers. Provide information on where the materials can be accessed | What (materials) Describe any physical or informational materials used in the intervention, including those provided to participants or used in intervention delivery or in training of intervention providers. Provide information on where the materials can be accessed | No change |
| **5** | What (procedures)Describe each of the procedures, activities, and/or processes used in the intervention, including any enabling or support activities | What (procedures) Describe whether the intervention was embedded in the core curriculum (e.g. within lectures, seminars, workshops), assessment design, adaption to pedagogy, or outside of the core curriculum. If the intervention is curriculum embedded, describe whether the intervention was credited or non-credited, compulsory or optional, assessed or non-assessed (add details of assessment if relevant) | Terminology adapted for education context. Detail added regarding context of intervention e.g. credited, optional, assessment. |
| **6** | Who providedFor each category of intervention provider (for example, psychologist, nursing assistant), describe their expertise, background and any specific training given | Who provided  Describe intervention provider, for example, lecturer, teaching assistant, service support staff. | Acknowledges types of provider in education context. Providers expertise and background is reflected in item 11. |
| **7** | HowDescribe the modes of delivery (such as face to face or by some other mechanism, such as internet or telephone) of the intervention and whether it was provided individually or in a group | How Describe the modes of delivery (such as face to face or by some other mechanism, such as internet or telephone) of the intervention and whether it was provided individually or in a group. | Acknowledges types of modules and how they are accessed. |
| **8** | WhereDescribe the type(s) of location(s) where the intervention occurred, including any necessary infrastructure or relevant features | Where Describe the type(s) of location(s) where the intervention occurred (e.g. classroom, lecture theatre, student union space, online), including any necessary infrastructure or relevant features (e.g. size of space). | Examples are given in this context. |
| **9** | When and how muchDescribe the number of times the intervention was delivered and over what period of time including the number of sessions, their schedule, and their duration, intensity or dose | When and how much Describe the number of times the intervention was delivered and over what period of time including the number of sessions, session duration, over what time period (e.g. 10 weekly, 2-hour sessions over 1 semester) | Terminology adapted for education context |
| **10** | ModificationsIf the intervention was modified during the course of the study, describe the changes (what, why, when, and how) | Modifications If the intervention was modified during the course of the study, describe the changes (what, why, when, and how) and reasons for the changes | A clause added to encourage description for reasons for any changes. |
| **11** | How well (planned)If intervention adherence or fidelity was assessed, describe how and by whom, and if any strategies were used to maintain or improve fidelity, describe them | How well  Describe adherence to intervention i.e. how many sessions students attended and number of students who dropped out. Describe providers education expertise (e.g. years of teaching), background and any specific training given (teacher training and/or training specific to deliver the intervention) to reflect fidelity. Describe any form of observation and assessment of the intervention delivery. | Terminology adapted for education context. Includes provider training and observation as means of fidelity. |
| **12** | How well (actual)If intervention adherence or fidelity was assessed, describe the extent to which the intervention was delivered as planned | n/a | n/a as covered in item 11. |

## Supplementary S2: CLOSER and CIDER checklists for after Stage 2 expert team meeting (ready for Stage 3 Delphi Round 1)

| **The CLOSER checklist for Delphi round 1** |
| --- |
| 1a. Abstract: Structured summary of design, methods, results, and conclusions. |
| 2a. Introduction (Background and objectives): Background and explanation of rationale. *NOTE: Within the checklist, we will suggest referring back to the CIDER checklist for further guidance here (no need to look back for the purposes of this survey, thank you,)* |
| 2b. Introduction (Background and objectives): Specific objectives or hypotheses. |
| 3a. Methods (study design): Description of study design (such as RCT, non-RCT, or pre-post without control). |
| 3b. Methods (Study Design): State any changes to methods after the study started (such as eligibility criteria), with reasons. |
| 4a. Methods (Participants): Eligibility criteria for participants at a group (e.g. cohort type) and individual level. |
| 4b. Methods (Participants): Settings and locations where the data were collected. *NOTE: Within the checklist, we will suggest referring back to the CIDER checklist for further guidance here (no need to look back for the purposes of this survey, thank you)* |
| 4c. Methods (Participants): Dates defining the periods of recruitment and follow-up. |
| 4d. Methods (Participants): Report methods of recruitment. Provide details on whether eligible participants who met the eligibility criteria were given equal opportunity to participate. |
| 5a. Methods (Interventions): Details of intervention with sufficient details to allow replication. *NOTE: Within the checklist, we will suggest referring back to the CIDER checklist for further guidance here (no need to look back for the purposes of this survey, thank you,)* |
| 5b. Methods (Interventions): Where there is a control condition(s), provide details of the control with sufficient details to allow replication. |
| 6a. Methods (Outcomes): Completely defined pre-specified primary and secondary outcome measures, including how and when they were assessed. Report whether they were validated and/or reasons for including unvalidated measures. |
| 6b. Methods (Outcomes): Any changes to outcomes after the study commenced, with reasons. |
| 7a. Methods (Sample size): How sample size was determined. |
| **The following section of the CLOSER checklist splits off according to study design.**  **The item below refers to non-RCTs and pre-post without controls.** |
| 8aa Methods (Bias): Identify potential sources of bias. Describe any efforts to address potential sources of bias. |
| **The following section of the CLOSER checklist splits off according to study design. The item below refers to RCTs.** |
| 8ab Methods (Sequence generation): Method used to generate the random allocation sequence. |
| 8b Methods (Sequence generation): Type of randomisation (e.g., computer-generated, coin flip, quasi-random etc); details of any restriction (such as blocking and block size). |
| 9. Methods (Allocation concealment mechanism): Mechanism used to implement the random allocation sequence (such as sequentially numbered containers), describing any steps taken to conceal the sequence until interventions were assigned. |
| 10. Methods (Implementation): Who generated the random allocation sequence, who enrolled participants, and who assigned participants to interventions. |
| 11a. Methods (Blinding): If done, who was blinded after assignment to interventions (for example, participants, providers, those assessing outcomes) and how. |
| 11b. Methods (Blinding): If relevant, description of the similarity of interventions. |
| **The remaining part of the CLOSER checklist resumes to refer to all study designs.** |
| 12a. Methods (Statistical methods): Statistical methods used to compare groups for primary and secondary outcomes. |
| 12b. Methods (Statistical methods): Methods for additional analyses, such as subgroup analyses and adjusted analyses. State reason if subgroup analyses are not possible or not appropriate. |
| 13a. Results (Participant flow): For each group, the numbers of participants who were assigned to each group, received the intended intervention, and were analysed for the primary outcome. |
| 13b. Results (Participant flow): For each group (where relevant), losses and exclusions (after randomisation or group allocation where relevant), together with reasons. |
| 14. Results (baseline data): A table showing baseline demographic characteristics for each group. |
| 15. Results (Numbers analysed): For each group (where applicable) and overall cohort, report the response rate and attrition rate. |
| 16a. Result (Outcomes and estimation): For each primary and secondary outcome, results for each group, at each time point. Report the estimated effect size and its precision (such as a 95% confidence interval). If there are ≥2 groups and ≥2 time points, an analysis that takes into consideration both time and group should be reported. |
| 16b. Results (Outcomes and estimation): For binary outcomes, the presentation of both absolute and relative effect sizes is recommended. |
| 17. Results (Ancillary analyses): Results of any other analyses performed, including subgroup analyses and adjusted analyses, distinguishing pre-specified from exploratory. |
| 18. Results (Harms): All important harms or unintended effects in each group. |
| 19. Discussion (Limitations): Study limitations, addressing sources of potential bias, imprecision, and, if relevant, multiplicity of analyses. |
| 20. Discussion (Generalisability): Generalisability (external validity, applicability) of the study findings. |
| 21. Results (Interpretation): Interpretation consistent with results, balancing benefits and harms, and considering other relevant evidence. |
| 22. Other information (Registration): Registration number and name of study registry. |
| 23. Other information (Protocol): Where the full study protocol can be accessed, if available. |
| 24. Other information (Funding): Sources of funding and other support, role of funders. |
| 25. Other information (Data repository ): Report the use of any data repository. |

| **The CIDER checklist for Delphi Round 1** |
| --- |
| 1. Brief name: Provide the name or a phrase that clearly describes the intervention. |
| 2. Why: Describe the rationale for intervention development with reference to theory or observations. |
| 3. What (materials): Describe learning objectives where appropriate. Describe any physical or informational materials used in the intervention, including those provided to participants or used in intervention delivery or training of intervention providers. Provide information on where the materials can be accessed. |
| 4. What (procedures): Describe whether the intervention was embedded in the core curriculum (e.g. within lectures, seminars, workshops), assessment design, adaption to pedagogy, or outside of the core curriculum. If the intervention is curriculum embedded, describe whether the intervention was credited, compulsory, and/or assessed (add details of assessment if relevant). Describe the nature of any ‘home practice’ students were given. |
| 5. Intervention developer: Describe who designed the intervention and their professional background and expertise. Report whether the person who designed the intervention delivered the intervention and/or trained those delivering the intervention. |
| 6. Who (for): Describe who the intervention was developed for and who could access it. Report if the intervention was open to all students across the institution or for a specific cohort. For data collected at baseline, report the participant’s demographics such as age, gender and ethnicity. |
| 7. Incentives for participants: Describe any incentives offered to student participants. |
| 8. Who (provided): Describe the intervention provider. Comment on any prior relationship between students and providers. Describe their expertise, professional discipline, background and any specific training given. |
| 9. How: Describe the modes of delivery (such as face-to-face, including lectures, seminars or workshops; or by some other mechanism, such as the internet or telephone) of the intervention and whether it was provided individually or in a group. Report the ratio of students to instructors. |
| 10a. Where (Setting): Describe the setting of the intervention, for example, the nature of the institution (urban or rural; school, further education, higher education; campus, commuter, distance-learning university). Report the size of the institution, i.e. total student population—comment on whether the study was conducted across one or multiple institutions. |
| 10b. Where (Location): Describe the type(s) of location(s) where the intervention occurred (e.g. classroom, lecture theatre, online, workplace placement, off-campus visits, green space), including any necessary infrastructure or relevant features (e.g. size of space). |
| 11. When and how much: Describe the number of times the intervention was delivered and over what period of time including the number of sessions (and for any home practice where relevant). If the content is self-paced, report how long the content was designed to be completed in (e.g. 30 hours across 2 semesters) and what guidance was given to the students. Comment on the time of year/semester the intervention was delivered, including any other time-appropriate information, e.g., delivered close to the exam period. |
| 12. Modifications: If the intervention was modified during the course of the study, describe the changes (what, why, when, and how) and the reasons for the changes. |
| 13a. How well (uptake): Describe the student’s attendance, including how this was assessed and by whom. Describe any strategies that were used to facilitate attendance. |
| 13b How well (observation or feedback): Describe any form of observation and assessment of the intervention delivery, i.e. teacher/trainer observation and student satisfaction. |
| 13c. How well (delivered as planned) Describe the extent to which the following features were delivered as planned (i.e. originally designed): who the intervention was for (was the sample representative of the student cohort in terms of measured demographics), intervention materials, procedures, who instructed/delivered the sessions, mode of delivery, location, the number of sessions, their frequency, timing and duration of the intervention. |
| 13d. How well (direct measurements): Report whether the outcome of the intervention was directly measured, e.g. in a stress management intervention, was stress measured pre and post-intervention. |

## Supplementary S3: CLOSER and CIDER checklist feedback from Stage 3 Delphi Round 1 (ready for Stage 4 Delphi Round 2)

| **CLOSER checklist survey responses, item adaptions and authorship team response to feedback from Delphi Round 1** | | | | | |
| --- | --- | --- | --- | --- | --- |
| Section/Topic | Item No | **Most common survey response from Delphi round 1 (mode)** | **CLOSER checklist item before Delphi round 1** | **CLOSER checklist item after Delphi round 1 feedback** | **Notes on our response to Delphi 1 feedback** |
| **Abstract** | 1a | Essential | Structured summary of design, methods, results, and conclusions. | Structured summary of design, methods, results, and conclusions. | No feedback for this item. |
| **Introduction: Background and objectives** | 2a | Essential | Background and explanation of rationale. Refer to item 3 in the CIDER checklist. | Background and explanation of rationale for study. | The reference to the CIDER checklist here was confusing, therefore it has been removed. This item is to encourage the use of an introduction section that provides background and rationale for the study. We refer to the CIDER checklist in item 5a only. |
| **Introduction: Background and objectives** | 2b | Essential | Specific objectives or hypotheses. | Succinctly state: (i) overall aim of the intervention, (ii) any specific objective(s), (iii) study hypotheses or research questions. | We have adapted the language in this item for clarity.  In our accompanying manuscript, we will justify why we recommend the inclusion of aims, objectives and hypotheses in quantitative research. We will also provide examples. |
| **Methods: Study design** | 3a | Essential | Description of study design (such as RCT, non-RCT, or pre-post without control, cross-sectional). | Description of study design (such as RCT, non-RCT, or pre-post without control, cross-sectional). | Whilst we appreciate some challenges with RCTs in education intervention research, please note these guidelines cover many designs in quantitative research including longitudinal (RCTs, non-RCTs, pre-post without control) and cross-sectional studies. We will define all these types of study designs in our manuscript. |
| **Methods: Study design** | 3b | Essential | State any changes to methods after the study started (such as eligibility criteria), with reasons. | State any changes to methods after the study started (such as eligibility criteria), with reasons. | No feedback for this item. |
| **Methods: Study design** | 3c | Essential |  | State whether ethics has been obtained for this research. | This new item regarding ethics has been added. |
| **Methods: Eligibility criteria** | 4a | Essential | Eligibility criteria for participants at a group (e.g. cohort type) and individual level. | State eligibility criteria for participants at (i) group (e.g., cohort type) level. (ii) individual level (e.g., demographic characteristics). | We will consider comments regarding this item when providing further explanations and examples of the checklist item in our accompanying manuscript, for example, defining ‘individual level’. |
| **~~Methods: Participants~~** | - | Essential with edits | Settings and locations where the data were collected. Please refer to CIDER guidelines. | ITEM REMOVED | We have removed this item as it is covered in the CIDER checklist which is referred to in item 5a. |
| **Methods: Recruitment** | 4b | Essential | Dates defining the periods of recruitment and follow-up. | Report dates defining the periods of recruitment and follow-up. | No feedback for this item. |
| **Methods: Recruitment** | 4c | Essential | Report methods of recruitment. Provide details on whether eligible participants who met the eligibility criteria were given equal opportunity to participate. | (i) Report methods of recruitment. (ii) Provide details on whether eligible participants who met the eligibility criteria were given equal opportunity to participate. | No feedback for this item. |
| **Methods: Interventions** | 5a | Essential | Details of intervention with sufficient details to allow replication. Please refer to CIDER checklist. | Summarise the intervention with sufficient details to implement in other contexts. Please use the CIDER checklist here. | We have replaced the word ‘replication’ with ‘implement in other contexts’. We will support this with further explanations and examples of the checklist item in our accompanying manuscript. |
| **Methods: Interventions** | 5b | Essential | Where there is a control condition(s), provide details of the control with sufficient details to allow replication. | Where there is a control condition(s), provide sufficient details to implement in other contexts. | We have removed the word ‘replication’ in this item too. |
| **Methods: Outcomes** | 6a | Essential | Completely defined pre-specified primary and secondary outcome measures, including how and when they were assessed. Report whether they were validated and/or reasons for including unvalidated measures. | Outline pre-specified primary and secondary outcome measures, including (i) how and when they were assessed. (ii) report whether they were validated and/or reasons for including unvalidated measures. | In our accompanying manuscript, we will highlight the need to specify what population the measures were validated in.  Please note these checklists are for quantitative research only. |
| **Methods: Outcomes** | 6b | Essential | Any changes to outcomes after the study commenced, with reasons. | Report any changes to outcomes after the study commenced, with reasons. | No feedback for this item. |
| **Methods: Sample size** | 7a | Essential | How sample size was determined. | State how the sample size was determined. | Please note we will refer to power calculations and other methods in our explanation of this item in our accompanying manuscript. Examples will be provided. |
| **Methods: Bias** | 7b | Essential | For RCTs continue here. For non-RCTs, pre-post without controls, or cross-sectional studies, please skip to 12a.  Identify potential sources of bias. Describe any efforts to address potential sources of bias. Explain here any items in the quality assessment tool that could not be met. | Identify potential sources of bias. Indicate any efforts to address potential sources of bias. | We have amended this item so it is appropriate for all study designs.  This item is particularly relevant for non-randomised study designs and will be considered further in the accompanying manuscript. |
| **Methods: Sequence generation** | 8a | Essential | Method used to generate the random allocation sequence. | *For RCTs continue here. For non-RCTs, pre-post without controls, or cross-sectional studies, please skip to 12a.*  State the method used to generate the random allocation sequence. | No feedback for this item. |
| **Methods: Sequence generation** | 8b | Essential | Type of randomisation (e.g., computer generated, coin flip, quasi random etc); details of any restriction (such as blocking and block size). | Report the type of randomisation (e.g., computer-generated, coin flip, quasi-random etc); details of any restriction (such as blocking and block size). | No feedback for this item. |
| **Methods: Allocation concealment mechanism** | 9 | Desirable | Mechanism used to implement the random allocation sequence (such as sequentially numbered containers), describing any steps taken to conceal the sequence until interventions were assigned. | Report the mechanism used to implement the random allocation sequence (such as sequentially numbered containers), describing any steps taken to conceal the sequence until interventions were assigned. | No feedback for this item. |
| **Methods: Implementation** | 10 | Essential | Who generated the random allocation sequence, who enrolled participants, and who assigned participants to interventions. | Report (i) who generated the random allocation sequence, (ii) who enrolled participants, and (iii) who assigned participants to interventions. | No feedback for this item. |
| **Methods: Blinding** | 11a | Essential | If done, who was blinded after assignment to interventions (for example, participants, providers, those assessing outcomes) and how. | If done, report who was blinded after assignment to interventions (for example, participants, providers, those assessing outcomes) and how. | No feedback for this item. |
| **Methods: Blinding** | 11b | Essential | If relevant, description of the similarity of interventions. | If relevant, provide a description of the similarity of interventions. | No feedback for this item. |
| **Methods: Statistical methods** | 12a | Essential | Statistical methods used to compare groups for primary and secondary outcomes. | Report statistical methods used to compare groups for primary and secondary outcomes. | We will provide clear detail regarding what statistics to report in our accompanying manuscript. |
| **Methods: Statistical methods** | 12b | Essential | Methods for additional analyses, such as subgroup analyses and adjusted analyses. State reason if subgroup analyses are not possible or not appropriate. | Report methods for additional analyses, such as subgroup analyses and adjusted analyses. State reason if subgroup analyses are not possible or not appropriate. | No feedback for this item. |
| **Results: Participant flow (a diagram is strongly recommended)** | 13a | Essential | For each group, the numbers of participants who were assigned to each group, received intended intervention, and were analysed for the primary outcome. | For each group, report the numbers of participants (i) who were assigned to or participated in each group, (ii) received intended intervention, and (ii) were analysed for the primary outcome. | We have added the phrasing “participated in” to accommodate for multiple study designs. |
| **Results: Participant flow (a diagram is strongly recommended)** | 13b | Essential | For each group (where relevant), losses and exclusions (after randomisation or group allocation where relevant), together with reasons. | For each group (where relevant), report losses and exclusions (after randomisation or group allocation where relevant), together with reasons. | No feedback for this item. |
| **Results: Baseline data** | 14 | Essential | A table showing baseline demographic characteristics for each group. | Provide a table showing baseline demographic characteristics for each group. | No feedback for this item. |
| **Results: Numbers analysed** | 15 | Essential | For each group (where applicable) and overall cohort, report the response rate and attrition rate. | For each group (where applicable) and overall cohort, report the response rate and attrition rate. | Please note that student engagement is covered in the CIDER checklist. |
| **Results: Outcomes and estimation** | 16a | Essential | For each primary and secondary outcome, results for each group, at each timepoint. Report the estimated effect size and its precision (such as 95% confidence interval). If there are ≥2 groups and ≥2 timepoints, an analysis that takes into consideration both time and group should be reported. | For each primary and secondary outcome, report (i) the results for each group, at each timepoint. (ii) the estimated effect size and its precision (such as 95% confidence interval). | We have removed some of the content from this checklist to simplify, we will re-add content to the explanation of this item in our accompanying manuscript. |
| **Results: Outcomes and estimation** | 16b | Essential | For binary outcomes, presentation of both absolute and relative effect sizes is recommended. | For binary outcomes, present both absolute and relative effect sizes is recommended. | No feedback for this item. |
| **Results: Ancillary analyses** | 17 | Essential | Results of any other analyses performed, including subgroup analyses and adjusted analyses, distinguishing pre-specified from exploratory. | Report the results of any other analyses performed, including subgroup analyses and adjusted analyses. | We have simplified this item and will provide further explanations and examples in our accompanying manuscript. |
| **Results: Harms** | 18 | Essential | All important harms or unintended effects in each group. | Report any unintended harms or effects in each group. Indicate how this was handled. | We have expanded this item to request researchers to detail how unintended harms or effects were handled. We will provide further explanation and examples in our accompanying manuscript. |
| **Discussion: Limitations** | 19 | Essential | Study limitations, addressing sources of potential bias, imprecision, and, if relevant, multiplicity of analyses. | State study limitations. | We have simplified this item and will provide further explanation and examples in our accompanying manuscript. |
| **Discussion: Generalisability** | 20 | Essential | Generalisability (external validity, applicability) of the study findings. | Comment on the ability of study findings to be implemented in other contexts. | We have adjusted the language of this item so it is appropriate for educational research. |
| **Discussion: Interpretation** | 21 | Essential | Interpretation consistent with results, balancing benefits and harms, and considering other relevant evidence. | State interpretation of results, balancing benefits and harms and considering other relevant evidence. | No feedback for this item. |
| **Other information: Registration** | 22 | Desirable | Registration number and name of study registry. | State registration number and name of study registry. | No feedback for this item. |
| **Other information: Protocol** | 23 | Desirable | Where the full study protocol can be accessed, if available. | Report where the full study protocol can be accessed, if available. | No feedback for this item. |
| **Other information: Funding** | 24 | Desirable | Sources of funding and other support, role of funders. | State the sources of funding and other support, the role of funders. | No feedback for this item. |
| **Other information: Data repository** | 25 | Desirable | Report use of any data repository. | Report the use of any data repository. | No feedback for this item. |

After receiving feedback from Stage 3 Delphi Round 1, we revised the CIDER checklist by breaking down the content of each item and modifying the language used across items. We also reordered and relabelled the CIDER checklist item headings based on the received feedback. The changes were from and to the following:

| **Checklist item labels before Delphi 1 feedback** | **Checklist item labels after Delphi 1 feedback** |
| --- | --- |
| 1. Brief name | 1. Brief title of intervention. |
| 1. Why | 2. Aim and objectives of intervention |
| 1. What (materials) | 3. Rationale for intervention |
| 1. What (procedures) | 4. Participants |
| 1. Intervention developer | 5a. Intervention provider(s) |
| 1. Who for | 5b. Intervention developer(s) |
| 1. Incentives for participants | 6a. Institutional setting |
| 1. Who provided | 6b. Intervention location |
| 1. How | 7. Intervention timing and duration |
| 10a. Where: Setting | 8a. Delivery – Modes and content |
| 10.b Where: Location | 8b. Delivery – Procedures |
| 11. When and how much | 9. Materials |
| 12. Modifications | 10a. Evaluation – Attendance |
| 13a. How well: uptake | 10b. Evaluation – Delivery |
| 13b. How well: observation or feedback | 10c. Evaluation – Intervention design |
| 13c. How well: delivered as planned | 10d. Evaluation – Outcome measurements |
| 13d. How well: direct measurements | 11. Modifications to intervention |
|  | 12. Incentives for participants |

| **CIDER checklist survey responses, item adaptions and authorship team response to feedback from Delphi Round 1** | | | |
| --- | --- | --- | --- |
| **Most common survey response from Delphi round 1** | **CIDER checklist item before Delphi round 1** | **CIDER checklist item after Delphi round 1 feedback** | **Notes on our response to Delphi 1 feedback** |
| Essential as is | **Brief name:** Provide the name or a phrase that clearly describes the intervention. | **1 Brief title of intervention:** Provide the name or a phrase that captures key 'essence' or intention of the intervention. | We have reworded this item for clarity. |
|  |  | **2 Aim and objectives of intervention:** Succinctly state: (i) overall aim of the intervention. (ii) objective(s) and/or intended learning outcome(s) of the intervention. | New item added in response to Delphi round 1 feedback. |
| Essential as is | **Why:** Describe the rationale for intervention development with reference to theory or observations. | **3 Rationale for intervention:** Report the purpose of intervention development with reference to relevant theory or observations. | In our accompanying manuscript with will explain this item further, e.g. researchers should provide justifications, and could critique other similar interventions etc. |
| Essential as is | Who ForDescribe who the intervention was developed for and who could access it. Report if the intervention was open to all students across the institution or for a specific cohort. For data collected at baseline, report the participant’s demographics such as age, gender and ethnicity. | **4 Participants:**  (i) Report who the intervention was developed for. (ii) Report if the intervention was open to all participants across the institution or for a specific cohort. iii) Report any collected participant demographic data such as age, gender and ethnicity. | Language has been amended for suitability in education intervention research. |
| Essential as is | **Who provided**  Describe the intervention provider. Comment on any prior relationship between students and providers. Describe their expertise, professional discipline, background and any specific training given. | **5a Intervention provider(s):**  (i) Indicate who provided the intervention, e.g., state the name and/or title of instructor(s) and the education organisation providing the intervention. (ii) report the relationship between intervention provider and developer, (iii) Comment on any prior relationship between participants and instructors. (iv) Report the instructors’ expertise, professional discipline, background and any relevant training they have undertaken. | Language has been amended for suitability in education intervention research.  For example,  Providers= hosting education organisation or outside organisation.  Developer= those who designed the intervention.  Instructor= those who delivered the intervention to the students. |
| Essential as is | Intervention developer: Describe who designed the intervention and their professional background and expertise. Report whether the person who designed the intervention delivered the intervention and/or trained those delivering the intervention. | **5b Intervention developer(s):**  (i) Report who designed the intervention (developer[s]) and their professional background(s) and expertise. (ii) Report whether developer(s) were also involved in delivering the intervention (instructor[s]) and/or trained the instructors. (iii) Report whether participants (particularly those with lived experience where relevant) were involved in co-creation of the intervention. If so, explain their involvement, if not, provide reasoning for this. | We have now adapted the checklist item to incorporate feedback.  Please note funding information is requested in the CLOSER checklist. |
| Essential as is | Where: SettingDescribe the setting of the intervention, for example, the nature of the institution (urban or rural; school, further education, higher education; campus, commuter, distance-learning university). Report the size of the institution, i.e. total student population—comment on whether the study was conducted across one or multiple institutions. | **6a Institutional setting:**  Indicate whether the intervention was conducted across one or multiple institutions. For each institution, report their nature and location, e.g., for example, the nature of the institution (i) Urban or rural; (ii) School, further education, higher education; (iii) Teaching on campus, online or both. Report (iv) the size of the institution, i.e. total student population. | There were some concerns regarding certain details of this item. We will justify why we have made these recommendations in our accompanying manuscript.  We will also discuss the importance of discussing cultural context when providing further explanations and examples of the checklist item in our accompanying manuscript.  We will encourage reporting of relevant demographics e.g., total institution student population with breakdown by full-time/part-time status; undergraduate versus postgraduate. |
| Essential as is | Where: LocationDescribe the type(s) of location(s) where the intervention occurred (e.g. classroom, lecture theatre, online, workplace placement, off-campus visits, green space), including any necessary infrastructure or relevant features (e.g. size of space). | **6b Intervention location:**  (i) Report the location(s) where the intervention occurred, e.g., classroom, lecture theatre, online, workplace placement, off-campus visits, green space. (ii) Include relevant infrastructure details, e.g., size of space, formal or informal layout. | We will consider comments regarding this item, e.g. replicability, when providing further explanations and examples of the checklist item in our accompanying manuscript. |
| Essential as is | When and how muchDescribe the number of times the intervention was delivered and over what period of time including the number of sessions (and for any home practice where relevant). If the content is self-paced, report how long the content was designed to be completed in (e.g. 30 hours across 2 semesters) and what guidance was given to the students. Comment on the time of year/semester the intervention was delivered, including any other time-appropriate information, e.g., delivered close to the exam period. | **7 Intervention timing and duration:**  Indicate: (i) The number of times the intervention was delivered, and over what period of time. (ii) The number of sessions (and any home practice where relevant). (iii) If the content was self-paced, how long the content was designed to be completed in (e.g. 30 hours across 2 semesters), and what guidance was given to the participants. (v) The time of year/semester the intervention was delivered, including any other time-appropriate information, e.g., delivered close to the exam period. | Language has been amended for suitability in education intervention research. |
| Essential as is | HowDescribe the modes of delivery (such as face-to-face, including lectures, seminars or workshops; or by some other mechanism, such as the internet or telephone) of the intervention and whether it was provided individually or in a group. Report the ratio of students to instructors. | **8a Delivery – Modes and content:**  (i) Briefly outline main delivery method(s) for the intervention, e.g. face-to-face lectures, seminars, workshops; online videos, discussion. (ii) State whether activities were individual or group-based; instructor-led or self-paced by participants. (iii) Provide an outline of main content for each session or activity. (iv) Report the ratio of participants to instructors. | We have been through and standardised language throughout regarding instructors. |
| Essential as is | What (procedures): Describe whether the intervention was embedded in the core curriculum (e.g. within lectures, seminars, workshops), assessment design, adaption to pedagogy, or outside of the core curriculum. If the intervention is curriculum embedded, describe whether the intervention was credited, compulsory, and assessed (add details of assessment if relevant). Describe the nature of any ‘home practice’ students were given. | **8b Delivery – Procedures:**  Indicate whether the intervention was: (i) Embedded in the core curriculum, e.g., formally taught, included in assessment design, aligned with pedagogical approach; or (ii) Run outside of the core curriculum. If the intervention was curriculum embedded, indicate whether the intervention was credited, compulsory, and/or assessed (add details of assessment if relevant). Report the nature of any ‘home practice’ participants were given. | Thank you for your comments here, we agree that a wider context is important, and detail is needed to take this into consideration. We will consider comments regarding this item when providing further explanations and examples of the checklist item in our accompanying manuscript. |
| Essential as is | What (materials): Describe any physical or informational materials used in the intervention, including those provided to participants or used in intervention delivery or training of intervention providers. Provide information on where the materials can be accessed. | **9 Materials**  Report any physical or informational materials used in the intervention, including those: (i) provided to participants; (ii) used in intervention delivery; (ii) used in training of intervention instructors. Indicate where the materials can be accessed. | There were concerns regarding how much information is in this item and lack of specificity. We will consider comments regarding this item when providing further explanation of the checklist item in our accompanying manuscript to make each feature of the item clear. We will also provide readers with an example. |
| Essential as is | **How well: uptake** Describe the student’s attendance, including how this was assessed and by whom. Describe any strategies that were used to facilitate attendance. | **10a Evaluation – Attendance:**  Indicate: (i) The number of participants who participated in the intervention. If the intervention included multiple sessions, indicate participant numbers for each one, and total of participants who completed the whole intervention, or dropped out mid-way. (ii) How attendance figures were recorded, and by whom. (iii) Any strategies that were used to facilitate attendance. | We have adapted this item based on Delphi 1 feedback.  Please note feedback from those delivering the intervention is incorporated in item 10b.  We expect that feedback regarding engagement will occur in item 10b too, we will make this explicit when providing further explanations and examples of the checklist item in our accompanying manuscript. |
| Essential as is | **How well: observation or feedback**  Describe any form of observation and assessment of the intervention delivery, i.e. teacher/trainer observation and student satisfaction. | **10b Evaluation – Delivery:**  (i) Report any form of observation or assessment of the intervention delivery, i.e., instructor observation, student satisfaction questionnaire. | This item has been merged with another evaluation item. |
| Essential as is | **How well: delivered as planned**  Describe the extent to which the following features were delivered as planned (i.e. originally designed): who the intervention was for (was the sample representative of the student cohort in terms of measured demographics), intervention materials, procedures, who instructed/delivered the sessions, mode of delivery, location, the number of sessions, their frequency, timing and duration of the intervention. | **10c Evaluation – Intervention design:**  Indicate the extent to which the intervention was delivered as planned with regard to: (i) Participants, e.g. whether they constituted a representative demographic sample of the institution’s student cohort.  (ii) Intervention materials. (ii) procedures. (iii) Instructors. (iv) Mode of delivery. (v) Location and setting. (vi) Number of sessions and their frequency. (vii) Timing and duration of the intervention. | We will consider comments regarding this item when providing further explanations and examples of the checklist item in our accompanying manuscript. For example, consideration of time/workload in ability to carry out intervention as intended. We will also highlight that tailoring of an intervention is likely, and that we are looking for transparency and justification for such tailoring i.e., not necessarily a judgement of ‘how well’ an intervention was delivered. |
| Essential as is | **How well: direct measurements**  Report whether the outcome of the intervention was directly measured, e.g. in a stress management intervention, was stress measured pre and post-intervention. | **10d Evaluation – Outcome measurements**  Indicate whether the outcome of the intervention was directly measured, e.g., in a stress management intervention, was stress measured pre and post-intervention. | The reporting of outcome measures and citations for these measures is requested in the CLOSER checklist. |
| Essential as is | ModificationsIf the intervention was modified during the course of the study, describe the changes (what, why, when, and how) and the reasons for the changes. | **11 Modifications to intervention:** If the intervention design was modified during the course of the study, indicate the changes (what, why, when, and how) and the reasons for the changes. | No feedback for this item. |
| Essential as is | Incentives for participantsDescribe any incentives offered to student participants. | **12 Incentives for participants:** Report any incentives offered to student participants. | Please note information regarding barriers to participation is included in the CLOSER checklist.  In our accompanying manuscript, we will suggest researchers add a justification for the incentive e.g., to increase participation or to improve grades. |

## Supplementary S4: CLOSER and CIDER checklist feedback from Stage 4 Delphi Round 2 (ready for Stage 5 final adjustments)

Following feedback received after Delphi Round 2, several adaptations were made to the item explanations and elaborations in our checklists:

- The term 'non-randomised control trial' was replaced with 'quasi-experimental' for clarity and accuracy.
- It was suggested that researchers should explicitly state from whom they received ethical approval and provide a reference number, thereby promoting transparency.
- The manuscript was adjusted to guide researchers in detailing their data collection methods and timeline.
- Acronyms were spelt out wherever possible in the checklists to enhance clarity.
- The manuscript was revised to include suggestions on potential statistical tests to cater to a range of quantitative methodologies.
- The term 'ethics' was substituted with 'ethical clearance' to provide clear guidance.
- It was clarified that precise recruitment dates are not necessary. Instead, researchers can provide the months or overall time period for context.
- Further elaboration was provided indicating that the job title/role is sufficient when detailing information about instructors.
- The term 'example' was replaced with 'e.g.' throughout the manuscript to maintain consistency.
- The elaboration encouraged researchers to report relevant demographics, including protected characteristics, to enrich understanding of the study population.
- Clear definitions of 'provider', 'instructor', and 'developer' were provided in the manuscript.
- When relevant, the inclusion of the university's location and the languages spoken there was recommended.
- The checklist was amended to note that instructors could also include peers and interventions co-created by students.
- The checklist was adjusted to suggest that researchers outline the structure of the academic year at the institution when detailing the timing of the intervention.
- The checklist was revised to advise researchers to report if the intervention was student-led and, if so, to detail the support provided by staff.
- If the intervention was conducted outside the standard curriculum, the elaboration was adjusted to recommend reporting on any incentives or promotional activities offered to encourage student attendance.
- If the intervention was student-led, it was recommended to detail any relevant training provided to the students, in addition to instructors.
- The checklist was streamlined by merging information regarding incentives (previously item 12 in CIDER) with the item concerning procedures (previously item 8b in CIDER).

| **CLOSER checklist survey responses from Delphi Round 2** | | | |
| --- | --- | --- | --- |
| Section/Topic | Item No | **CLOSER checklist item** | **N (%) respondents who approve item for inclusion in final checklist** |
| **Abstract** | 1a | Structured summary of design, methods, results, and conclusions. | 19 (100) |
| **Introduction: Background and objectives** | 2a | Background and explanation of rationale for study. | 18 (94.7) |
| **Introduction: Background and objectives** | 2b | Succinctly state: (i) overall aim of the intervention, (ii) any specific objective(s), (iii) study hypotheses or research questions. | 19 (100) |
| **Methods: Study design** | 3a | Description of study design (such as RCT, non-RCT, or pre-post without control, cross-sectional). | 17 (89.5) |
| **Methods: Study design** | 3b | State any changes to methods after the study started (such as eligibility criteria), with reasons. | 18 (94.7) |
| **Methods: Study design** | 3c | State whether ethics has been obtained for this research. | 16 (84.2) |
| **Methods: Eligibility criteria** | 4a | State eligibility criteria for participants at (i) group (e.g., cohort type) level. (ii) individual level (e.g., demographic characteristics). | 19 (100) |
| **Methods: Recruitment** | 4b | Report dates defining the periods of recruitment and follow-up. | 17 (89.5) |
| **Methods: Recruitment** | 4c | (i) Report methods of recruitment. (ii) Provide details on whether eligible participants who met the eligibility criteria were given equal opportunity to participate. | 18 (94.7) |
| **Methods: Interventions** | 5a | Summarise the intervention with sufficient details to implement in other contexts. Please use the CIDER checklist here. | 19 (100) |
| **Methods: Interventions** | 5b | Where there is a control condition(s), provide sufficient details to implement in other contexts. | 18 (94.7) |
| **Methods: Outcomes** | 6a | Outline pre-specified primary and secondary outcome measures, including (i) how and when they were assessed. (ii) report whether they were validated and/or reasons for including unvalidated measures. | 19 (100) |
| **Methods: Outcomes** | 6b | Report any changes to outcomes after the study commenced, with reasons. | 18 (94.7) |
| **Methods: Sample size** | 7a | State how the sample size was determined. | 19 (100) |
| **Methods: Bias** | 7b | Identify potential sources of bias. Indicate any efforts to address potential sources of bias. | 18 (94.7) |
| **Methods: Sequence generation** | 8a | *For RCTs continue here. For non-RCTs, pre-post without controls, or cross-sectional studies, please skip to 12a.*  State the method used to generate the random allocation sequence. | 19 (100) |
| **Methods: Sequence generation** | 8b | Report the type of randomisation (e.g., computer-generated, coin flip, quasi-random etc); details of any restriction (such as blocking and block size). | 19 (100) |
| **Methods: Allocation concealment mechanism** | 9 | Report the mechanism used to implement the random allocation sequence (such as sequentially numbered containers), describing any steps taken to conceal the sequence until interventions were assigned. | 14 (73.7) |
| **Methods: Implementation** | 10 | Report (i) who generated the random allocation sequence, (ii) who enrolled participants, and (iii) who assigned participants to interventions. | 16 (84.2) |
| **Methods: Blinding** | 11a | If done, report who was blinded after assignment to interventions (for example, participants, providers, those assessing outcomes) and how. | 18 (94.7) |
| **Methods: Blinding** | 11b | If relevant, provide a description of the similarity of interventions. | 17 (89.5) |
| **Methods: Statistical methods** | 12a | Report statistical methods used to compare groups for primary and secondary outcomes. | 19 (100) |
| **Methods: Statistical methods** | 12b | Report methods for additional analyses, such as subgroup analyses and adjusted analyses. State reason if subgroup analyses are not possible or not appropriate. | 18 (94.7) |
| **Results: Participant flow (a diagram is strongly recommended)** | 13a | For each group, report the numbers of participants (i) who were assigned to or participated in each group, (ii) received intended intervention, and (ii) were analysed for the primary outcome. | 19 (100) |
| **Results: Participant flow (a diagram is strongly recommended)** | 13b | For each group (where relevant), report losses and exclusions (after randomisation or group allocation where relevant), together with reasons. | 18 (94.7) |
| **Results: Baseline data** | 14 | Provide a table showing baseline demographic characteristics for each group. | 18 (94.7) |
| **Results: Numbers analysed** | 15 | For each group (where applicable) and overall cohort, report the response rate and attrition rate. | 19 (100) |
| **Results: Outcomes and estimation** | 16a | For each primary and secondary outcome, report (i) the results for each group, at each timepoint. (ii) the estimated effect size and its precision (such as 95% confidence interval). | 18 (94.7) |
| **Results: Outcomes and estimation** | 16b | For binary outcomes, present both absolute and relative effect sizes is recommended. | 18 (94.7) |
| **Results: Ancillary analyses** | 17 | Report the results of any other analyses performed, including subgroup analyses and adjusted analyses. | 19 (100) |
| **Results: Harms** | 18 | Report any unintended harms or effects in each group. Indicate how this was handled. | 19 (100) |
| **Discussion: Limitations** | 19 | State study limitations. | 19 (100) |
| **Discussion: Generalisability** | 20 | Comment on the ability of study findings to be implemented in other contexts. | 18 (94.7) |
| **Discussion: Interpretation** | 21 | State interpretation of results, balancing benefits and harms and considering other relevant evidence. | 19 (100) |
| **Other information: Registration** | 22 | State registration number and name of study registry. | 18 (94.7) |
| **Other information: Protocol** | 23 | Report where the full study protocol can be accessed, if available. | 19 (100) |
| **Other information: Funding** | 24 | State the sources of funding and other support, the role of funders. | 19 (100) |
| **Other information: Data repository** | 25 | Report the use of any data repository. | 19 (100) |

| **CIDER checklist survey responses from Delphi Round 2** | |
| --- | --- |
| **CIDER checklist item** | **N (%) respondents who approve item for inclusion in final checklist** |
| **1 Brief title of intervention:** Provide the name or a phrase that captures key 'essence' or intention of the intervention. | 18 (94.7) |
| **2 Aim and objectives of intervention:** Succinctly state: (i) overall aim of the intervention. (ii) objective(s) and/or intended learning outcome(s) of the intervention. | 19 (100) |
| **3 Rationale for intervention:** Report the purpose of intervention development with reference to relevant theory or observations. | 18 (94.7) |
| **4 Participants:**  (i) Report who the intervention was developed for. (ii) Report if the intervention was open to all participants across the institution or for a specific cohort. iii) Report any collected participant demographic data such as age, gender and ethnicity. | 18 (94.7) |
| **5a Intervention provider(s):**  (i) Indicate who provided the intervention, e.g., state the name and/or title of instructor(s) and the education organisation providing the intervention. (ii) report the relationship between intervention provider and developer, (iii) Comment on any prior relationship between participants and instructors. (iv) Report the instructors’ expertise, professional discipline, background and any relevant training they have undertaken. | 17 (89.5) |
| **5b Intervention developer(s):**  (i) Report who designed the intervention (developer[s]) and their professional background(s) and expertise. (ii) Report whether developer(s) were also involved in delivering the intervention (instructor[s]) and/or trained the instructors. (iii) Report whether participants (particularly those with lived experience where relevant) were involved in co-creation of the intervention. If so, explain their involvement, if not, provide reasoning for this. | 17 (89.5) |
| **6a Institutional setting:** Indicate whether the intervention was conducted across one or multiple institutions. For each institution, report their nature and location, e.g., for example, the nature of the institution (i) Urban or rural; (ii) School, further education, higher education; (iii) Teaching on campus, online or both. Report (iv) the size of the institution, i.e. total student population. | 17 (89.5) |
| **6b Intervention location:** (i) Report the location(s) where the intervention occurred, e.g., classroom, lecture theatre, online, workplace placement, off-campus visits, green space. (ii) Include relevant infrastructure details, e.g., size of space, formal or informal layout. | 19 (100) |
| **7 Intervention timing and duration:** Indicate: (i) The number of times the intervention was delivered, and over what period of time. (ii) The number of sessions (and any home practice where relevant). (iii) If the content was self-paced, how long the content was designed to be completed in (e.g. 30 hours across 2 semesters), and what guidance was given to the participants. (v) The time of year/semester the intervention was delivered, including any other time-appropriate information, e.g., delivered close to the exam period. | 18 (94.7) |
| **8a Delivery – Modes and content:**  (i) Briefly outline main delivery method(s) for the intervention, e.g. face-to-face lectures, seminars, workshops; online videos, discussion. (ii) State whether activities were individual or group-based; instructor-led or self-paced by participants. (iii) Provide an outline of main content for each session or activity. (iv) Report the ratio of participants to instructors. | 18 (94.7) |
| **8b Delivery – Procedures:** Indicate whether the intervention was: (i) Embedded in the core curriculum, e.g., formally taught, included in assessment design, aligned with pedagogical approach; or (ii) Run outside of the core curriculum. If the intervention was curriculum embedded, indicate whether the intervention was credited, compulsory, and/or assessed (add details of assessment if relevant). Report the nature of any ‘home practice’ participants were given. | 18 (94.7) |
| **9 Materials** Report any physical or informational materials used in the intervention, including those: (i) provided to participants; (ii) used in intervention delivery; (ii) used in training of intervention instructors. Indicate where the materials can be accessed. | 17 (89.5) |
| **10a Evaluation – Attendance:**  Indicate: (i) The number of participants who participated in the intervention. If the intervention included multiple sessions, indicate participant numbers for each one, and total of participants who completed the whole intervention, or dropped out mid-way. (ii) How attendance figures were recorded, and by whom. (iii) Any strategies that were used to facilitate attendance. | 19 (100) |
| **10b Evaluation – Delivery:**  (i) Report any form of observation or assessment of the intervention delivery, i.e., instructor observation, student satisfaction questionnaire. | 18 (94.7) |
| **10c Evaluation – Intervention design:**  Indicate the extent to which the intervention was delivered as planned with regard to: (i) Participants, e.g. whether they constituted a representative demographic sample of the institution’s student cohort.  (ii) Intervention materials. (ii) procedures. (iii) Instructors. (iv) Mode of delivery. (v) Location and setting. (vi) Number of sessions and their frequency. (vii) Timing and duration of the intervention. | 19 (100) |
| **10d Evaluation – Outcome measurements**  Indicate whether the outcome of the intervention was directly measured, e.g., in a stress management intervention, was stress measured pre and post-intervention. | 19 (100) |
| **11 Modifications to intervention:** If the intervention design was modified during the course of the study, indicate the changes (what, why, when, and how) and the reasons for the changes. | 18 (94.7) |
| **12 Incentives for participants:** Report any incentives offered to student participants. | 18 (94.7) |
